# Statistic Copolymers Working as Growth Factor‐Binding Mimics of Fibronectin

**DOI:** 10.1002/advs.202200775

**Published:** 2022-05-15

**Authors:** Wenjing Zhang, Yueming Wu, Qi Chen, Haodong Zhang, Min Zhou, Kang Chen, Chuntao Cao, Han Guo, Jianrong Xu, Honglai Liu, Haodong Lin, Changsheng Liu, Runhui Liu

**Affiliations:** ^1^ State Key Laboratory of Bioreactor Engineering East China University of Science and Technology Shanghai 200237 China; ^2^ Key Laboratory for Ultrafine Materials of Ministry of Education Frontiers Science Center for Materiobiology and Dynamic Chemistry Research Center for Biomedical Materials of Ministry of Education School of Materials Science and Engineering East China University of Science and Technology Shanghai 200237 China; ^3^ Shanghai Synchrotron Radiation Facility (SSRF) Shanghai Advanced Research Institute Chinese Academy of Sciences Shanghai 201204 China; ^4^ Academy of Integrative Medicine Shanghai University of Traditional Chinese Medicine Shanghai 201203 China; ^5^ School of Chemistry and Molecular Engineering East China University of Science and Technology Shanghai 200237 China; ^6^ Department of Orthopedic Surgery Shanghai General Hospital Shanghai Jiao Tong University School of Medicine Shanghai 200080 China

**Keywords:** fibronectin mimicking, growth factor binding, statistic copolymers

## Abstract

Growth factors (GFs) play important roles in biological system and are widely used in tissue regeneration. However, their application is greatly hindered by short in vivo lifetime of GFs. GFs are bound to fibronectin dynamically in the extracellular matrix, which inspired the authors to mimic the GF binding domain of fibronectin and design GF‐binding amphiphilic copolymers bearing positive charges. The optimal amino acid polymer can bind to a variety of representative GFs, such as bone morphogenetic protein‐2 (BMP‐2) and TGF‐*β*1 from the transforming growth factor‐*β* superfamily, PDGF‐AA and PDGF‐BB from the platelet‐derived growth factor family, FGF‐10 and FGF‐21 from the fibroblast growth factor family, epidermal growth factor from the EGF family and hepatocyte growth factor from the plasminogen‐related growth factor family, with binding affinities up to the nanomolar level. 3D scaffolds immobilized with the optimal copolymer enable sustained release of loaded BMP‐2 without burst release and significantly enhances the in vivo function of BMP‐2 for bone formation. This strategy opens new avenues in designing GF‐binding copolymers as synthetic mimics of fibronectin for diverse applications.

## Graphical abstract

By mimicking the growth factor (GF) binding domain of fibronectin, FN III12‐14, statistic amino acid copolymers that have strong binding affinity to a variety of GFs are designed and identified, even comparable to that between the GFs and GFs’ receptor. These copolymers have obvious advantages in their high stability against enzymatic degradation, low price, and easy synthesis in large scale.

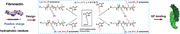

## 1 Introduction

Growth factors (GFs) play important roles in tissue repair and regeneration such as bone morphogenetic protein‐2 (BMP‐2) and vascular endothelial growth factor (VEGF) that induces osteogenesis and angiogenesis, respectively.^[^ ^1^ ^]^ However, GFs, like many proteins, are rapidly inactivated in vivo by protease. Consequently, the dosage of GFs is increased dramatically in clinical applications,^[^ ^2^ ^]^ which leads to high cost and the risk of severe side effects.^[^ ^3^ ^]^ Even though extensive studies have been conducted to protect GFs in vivo for tissue regeneration,^[^ ^4^ ^]^ it is still a formidable challenge to find a safe, generalizable and straightforward strategy to promote the stability and bioactivity of GFs and ultimately reduce the dosage of GFs in tissue regeneration.

**Figure 1:**
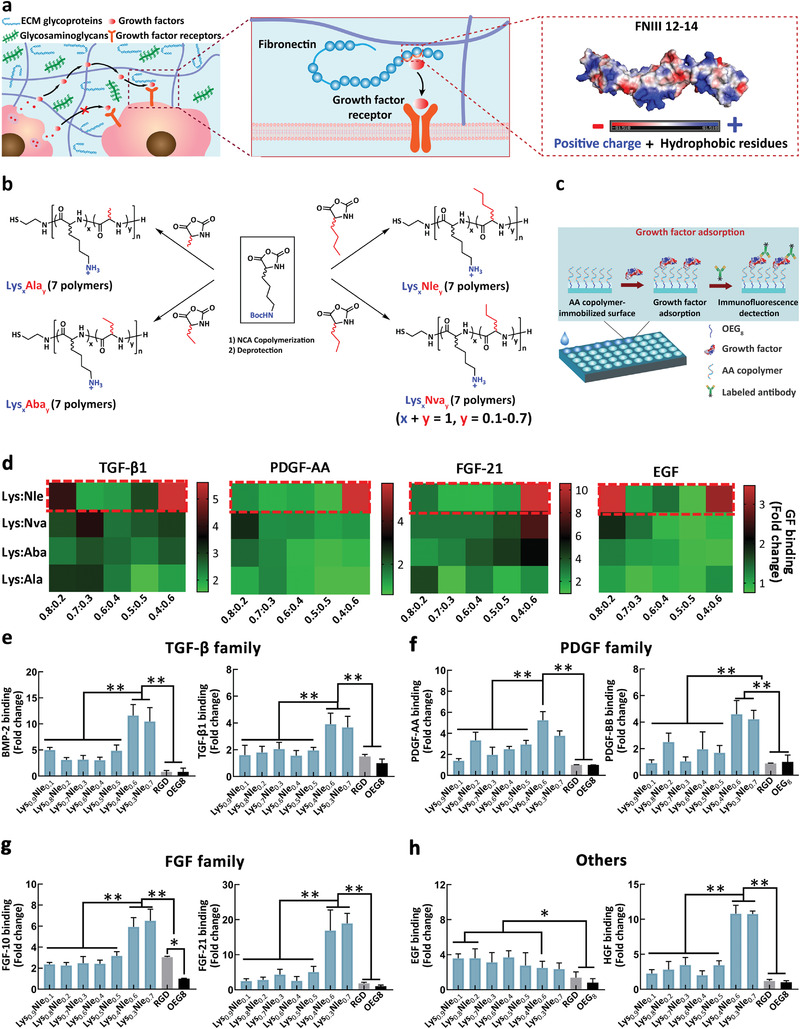
Fibronectin (FN) mimicking amino acid copolymer (AA copolymer) displaying growth factor (GF) binding function. a) FN is a ubiquitous ECM protein and possesses a binding domain, FNIII 12–14, toward diverse classes of GFs. Blue color represents the positive charge, red color refers to the negative charge and white color represents hydrophobic. b) The scheme for the synthesis of 28 FN mimicking amphiphilic AA copolymers. c) High throughput screening for GFs binding AA copolymers using an immunofluorescence assay. d) Heat map summarizing GF adsorption evaluation for AA polymer library (mean values from *n* = 2 replicates for each polymer). e‐h) GF binding ability to variable AA copolymers as evaluated from GFs adsorption study for transforming growth factor‐*β* (TGF‐*β*) superfamily (e), platelet‐derived growth factor (PDGF) family (f), fibroblast growth factor (FGF) family (g), and other families (h) (*p < 0.05, **p < 0.01, *n* = 5). Mean ± SD are shown.

This challenge encourages us to revisit the environment of GFs in the body wherein extracellular matrix (ECM) proteins bind to GFs directly, acting as a reservoir to protect GFs from protease degradation and burst release.^[^ ^5^ ^]^ Moreover, the ECM protein‐tethered GFs can still bind to their receptors, preventing endocytosis and resulting in prolonged, intense, and localized signaling by the ECM‐GF/GF receptor complex.^[^ ^6^ ^]^ Fibronectin (FN), as a ubiquitous ECM protein, possesses GF binding domains (GFBDs) toward diverse GFs (Figure 1a) such as TGF‐*β*1,^[^ ^7^ ^]^ BMP‐2,^[^ ^8^ ^]^ BMP‐6, and BMP‐7^[^ ^9^ ^]^ in transforming growth factor‐*β* (TGF‐*β*) superfamily, VEGF,^[^ ^10^ ^]^ and platelet‐derived growth factor (PDGF)‐BB^[^ ^11^ ^]^ in PDGF family, and hepatocyte growth factor (HGF).^[^ ^12^ ^]^ GFBDs of FN are also indispensable for cell survival and the downstream activation of GFs.^[^ ^11^ ^]^ Therefore, GFBDs of FN have been used especially for in vivo applications to protect GFs from proteolysis and to promote the function of GFs. For example, the extensively studied 12th to 14th type III repeats of FN (FN III12‐14), has been incorporated into hydrogels to protect VEGF and BMP‐2 and promote their functions for skin repair and bone regeneration, respectively.^[^ ^13^ ^]^ It also presents an approach for engineering the cellular microenvironment to enhance GF‐induced tissue healing that could be translated to human application, because all of the components except the engineered FN fragment are already used in clinic. However, the application and in vivo performance of GFBDs are greatly limited due to their innate shortcomings as peptides including poor in vivo stability, expensiveness, hard to produce in large quantity and inconvenient modification to scaffolds.

FN III12‐14 possesses highly promiscuous binding to most GFs within PDGF family and fibroblast growth factor family, and some GFs within TGF‐*β* superfamily and insulin‐like growth factor family.^[^ ^14^ ^]^ Precedent study reported that positively charged amino acids within the growth factor binding domain of fibronectin, such as lysine and arginine, are pivotal for growth factor binding, given that their substitution abolished the binding of growth factors.^[^ ^15^ ^]^ In addition, we examined the homopolymer poly‐Lys and found that the highly positively charged poly‐Lys showed weak growth factor binding ability (Figure S1, Supporting Information). We noticed that FN III12‐14 has conserved residues to have both hydrophobic residues and positive charges (Figure 1a).^[^ ^16^ ^]^ These observations imply that the overall amphiphilic structure and positive charges within FN III12‐14 govern the binding of FN III12‐14 to a large variety of GFs. This hypothesis encouraged us to design GF binding molecules by mimicking FN III12‐14 with positively charged amphiphilic amino acid copolymers (AA copolymers) that are composed of a mixture of a cationic residue (lysine (Lys)), and a hydrophobic residue (norleucine (Nle), norvaline (Nva), aminobutyric acid (Aba), or alanine (Ala)) (Figure 1b). The FN‐mimicking AA polymers are supposed to reversibly bind GFs and consequently regulate their bioactivity to enhance tissue regeneration and repair. It is noteworthy that both amino acids in our study are d/l racemic mixtures. The use of d amino acids enables the resulting polymers to have enhanced stability against enzymatic degradation.^[^ ^17^ ^]^ Using an immunofluorescent‐based high throughput screening, we identified the optimal AA copolymer that exerts strong binding to GFs from TGF‐*β* superfamily, PDGF family, fibroblast growth factor (FGF) family, and other families. Using BMP‐2 as a model, the optimal AA copolymer enables sustained release of loaded BMP‐2, without burst release, from 3D scaffolds and substantially enhanced in vivo activity in bone regeneration. Our study provides a generalizable strategy for developing GF binding copolymers that can have diverse applications.

## 2 Results and Discussion

### 2.1 High‐Throughput Screening of AA Copolymers for GF Binding

A cationic residue (racemic Lys) and a hydrophobic residue (racemic Nle, Nva, Aba, or Ala) at variable ratio were used to compose the heterochiral cationic‐amphiphilic AA copolymers. These copolymers were prepared from the ring‐opening polymerization on a mixture of two *α*‐amino acid *N*‐carboxyanhydride (NCA) monomers, a cationic NCA monomer (N*ε*‐*tert*‐butyloxycarbonyl‐d,l‐Lys NCA (Boc‐d,l‐Lys NCA)) and a hydrophobic NCA monomer (d,l‐Nle NCA, d,l‐Nva NCA, d,l‐Aba NCA, or d,l‐Ala NCA) (Figure 1b and Schemes S1–S4, Supporting Information). After treatment with trifluoroacetic acid in the presence of 5% triethylsilane to remove the protection groups, a series of thiol‐terminated copolymers at about 30 mer (DP = 28–35) and narrow dispersity (*Đ* = 1.10–1.20) were obtained with an incrementally increased ratio of the hydrophobic subunit from 10% to 70%, named Lys*_x_* Nle*_y_*, Lys*_x_* Nva*_y_*, Lys*_x_* Aba*_y_*, and Lys*_x_* Ala*_y_* (*x* + *y* = 1, *y* = 0.1–0.7) (Figures S2–S5 and Table S1, Supporting Information). NMR characterization confirmed that the ratio between cationic residue and hydrophobic residue within the copolymer chains was consistent to the feeding ratio of NCA monomers (Figures S6–S33, Supporting Information). One of the merits of these AA polymers, composed of racemic amino acid residues, lies in their high in vivo stability owing to their resistance to enzymatic degradation, as is demonstrated with Lys_0.4_Nle_0.6_ that shows no observable degradation after incubation with trypsin for two weeks (Figure S34, Supporting Information). As a comparison, a chiral AA homopolymer (l‐poly‐Lys) is almost completely degraded after incubation with trypsin within 77 h.^[^ ^18^ ^]^ In addition, the synthetic cost of AA copolymers is much cheaper than that of the conventional peptides (Table S2, Supporting Information).

GFs binding to above AA copolymers was assessed by a high‐throughput screening using copolymer immobilized 50 well glass slides, dye‐labeled GF antibody, and acquiring of fluorescence intensity after immunoassay (Figure 1c and Scheme S5, Supporting Information). An octaethylene glycol (OEG_8_) antifouling layer was used between the AA copolymers and the glass surface to suppress the fouling background signals and reveal the genuine GFs binding property of these AA copolymers. Those thiol‐terminated copolymers were covalently attached onto the glass surface by reacting with maleimide groups of heterobifunctional OEG_8_ (maleimide‐OEG_8_‐succinimidyl ester).^[^ ^19^ ^]^ The successful modification of the polymer to the surface was characterized by ellipsometry analysis to have increased surface thickness of 10.30 ± 0.83 nm (Figure S35a, Supporting Information). The successful modification of the polymer to the surface was also characterized by X‐ray photoelectron spectroscopy (XPS) analysis and confirmed by the appearance of N peak in XPS spectra (Figure S35b, Supporting Information).

Our initial screening results showed that, all 4 representative GFs from different protein families have strong binding to AA copolymer of Lys*_x_* Nle*_y_*, but not the other AA copolymers (Lys*_x_* Nva*_y_*, Lys*_x_* Aba*_y_*, and Lys*_x_* Ala*_y_*) (Figure 1d). We then further examined the ability of Lys*_x_* Nle*_y_* for binding to GFs including BMP‐2 and TGF‐*β*1 from the TGF superfamily (Figure 1e), PDGF‐AA and PDGF‐BB from PDGF family (Figure 1f), FGF‐10 and FGF‐21 from FGF family (Figure 1g), epidermal growth factor (EGF) from EGF family and HGF from the plasminogen‐related growth factor family (Figure 1h). Compared to OEG_8_ and RGD peptide, whom both have minimum binding to GFs, Lys_0.4_Nle_0.6_ displayed the strongest binding to all these GFs (Figure 1d–h). In addition, our experimental results showed that the homopolymer poly‐Lys showed only weak GF binding ability (Figure S1, Supporting Information), indicating the hydrophobic residue in the polymer is indispensable for GFs adsorption and the adsorption ability is not just the consequence of the presence of positive charges. Varying the chain length and chirality did not affect the binding ability of AA polymers (Figures S36 and S37, Supporting Information). Our results indicate that only optimal amphiphilic AA copolymer with specific composition can work as FN mimics to have strong binding to GFs.

### 2.2 Binding Affinity of GFs to Optimal AA Polymer

**Figure 2:**
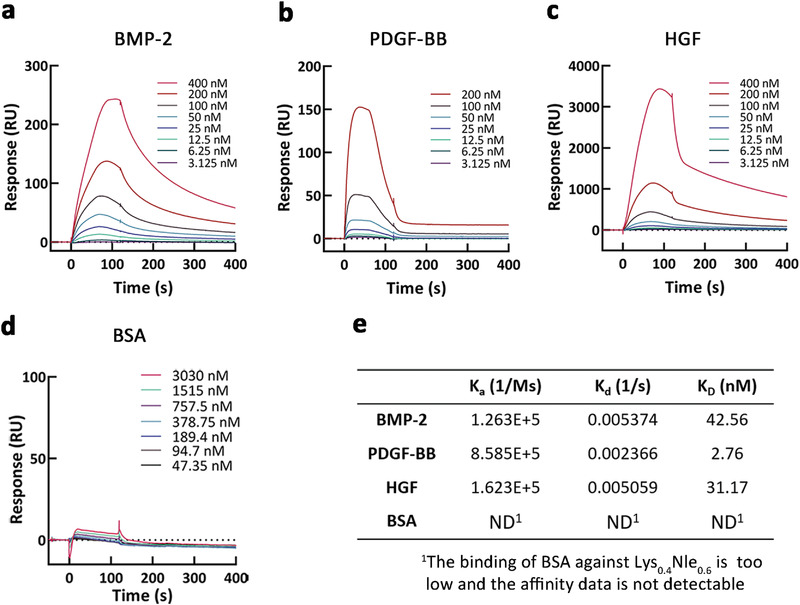
Growth factors binding affinity of the selected AA copolymer (Lys_0.4_Nle_0.6_) using surface plasmon resonance (SPR) analysis. a–d) SPR chip was functionalized with the selected AA copolymer (Lys_0.4_Nle_0.6_), and each protein was flown over the chip at indicated concentrations. e) GFs and Lys_0.4_Nle_0.6_ binding kinetics values determined from the experimental curve fits. ^1^ND means bovine serum albumin (BSA) has negligible binding to Lys_0.4_Nle_0.6_ as is not detectable.

We also measured the binding affinity between Lys_0.4_Nle_0.6_ and GFs (BMP‐2, PDGF‐BB, and HGF) using surface plasmon resonance (SPR) (Figure 2a–c) and calculated the binding affinity constants (*K*_D_, *K*_d_, and *K*_a_) via the obtained binding curves (Figure 2e). The specific dissociation constant (*K*_D_) between Lys_0.4_Nle_0.6_ and BMP‐2, PDGF‐BB, and HGF was 42.56 × 10^−9^, 2.76 × 10^−9^, and 31.17 × 10^−9^ m, respectively. Bovine serum albumin (BSA), for comparison, showed no specific interaction with Lys_0.4_Nle_0.6_ (Figure 2d). According to precedent report the binding affinity between fibronectin and BMP‐2 is 5.26× 10^−9^ m,^[^ ^9^ ^]^ which indicates that it is a valid and practical strategy to mimic fibronectin using statistic copolymers. The optimal GF binding copolymer, Lys_0.4_Nle_0.6_, was used for the following studies.

### 2.3 Binding Mode between Lys_0.4_Nle_0.6_ and GFs

**Figure 3:**
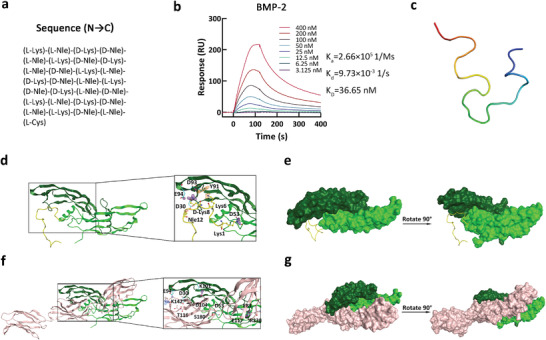
a) The amino acid sequence of the Lys‐Nle peptide. b) BMP‐2 binding affinity of the selected Lys‐Nle peptide using SPR analysis. BMP‐2 and Lys‐Nle peptide binding kinetics values determined from the experimental curve fits. c) 3D structure of Lys‐Nle peptide. d) The 3D binding model between BMP‐2 and Lys‐Nle peptide. The backbone of chain A of BMP‐2 is depicted as dark green cartoon, the backbone of chain B of BMP‐2 is depicted as green cartoon. The residues in BMP‐2 are colored in cyan. Lys‐Nle peptide is depicted as yellow cartoon. The residues in Lys‐Nle peptide are colored in yellow. e) The surface binding model between BMP‐2 and Lys‐Nle peptide. The surface of chain A of BMP‐2 is colored in dark green, the surface of chain B of BMP‐2 is colored in green. Lys‐Nle peptide is depicted as yellow cartoon. The lightblue dashes represent hydrogen bond interaction. There are salt bridges between the groups marked by the fog group. f) The 3D binding model between BMP‐2 and FNIII 12–14. The backbone of chain A of BMP‐2 is depicted as dark green cartoon, the backbone of chain B of BMP‐2 is depicted as green cartoon. The residues in BMP‐2 are colored in cyan. The backbone of FNIII 12–14 is depicted as pink cartoon. The residues in FNIII 12–14 are colored in pink. g) The surface binding model between BMP2 and FNIII 12–14. The surface of chain A of BMP‐2 is colored in dark green, the surface of chain B of BMP‐2 is colored in green and the surface of FNIII 12–14 is colored in pink. The lightblue dashes represent hydrogen bond interaction. There are salt bridges between the groups marked by the fog group.

To reveal the binding mode between the optimal AA polymer Lys_0.4_Nle_0.6_ and GFs, we synthesized a peptide, named Lys‐Nle peptide, with the same chain length and ratio of Lys:Nle as that of Lys_0.4_Nle_0.6_ (Figure 3a). The FDA‐approved and clinically used GF, BMP‐2, was used as a model in this study. The Lys‐Nle peptide has a BMP‐2 binding affinity of 36.65× 10^−9^ m (Figure 3b), comparable to 42.56× 10^−9^ m for Lys_0.4_Nle_0.6_ AA polymer, indicating that the Lys‐Nle peptide is valid in exploring the binding mechanism between Lys_0.4_Nle_0.6_ AA polymer and GFs. To search for the stable structure of the peptide, we performed a molecular docking search followed by an all‐atom, explicit water molecular dynamics (MD) simulations, using a clustering strategy for the MD trajectory (Figure S38, Supporting Information). The cluster center after the system's equilibrium was selected as the final stable structure of the peptide (Figure 3c).

Molecular docking was used to predict the binding sites and binding mode between the Lys‐Nle peptide and BMP‐2. In the backbone of Lys‐Nle peptide, we observed hydrogen bonding between residue Nle12 of Lys‐Nle peptide and Asp30 in chain A of BMP‐2. We also observed salt bridges between residue d‐Lys8 of Lys‐Nle peptide and Asp93/Glu94 in chain A of BMP‐2, *π*‐H interaction between Lys6 of Lys‐Nle peptide and residue Try91 in chain A of BMP‐2. In addition, Lys‐Nle peptide residue d‐Lys1 formed salt bridges with residue Asp53 in chain B of BMP‐2 (Figure 3d,e). For comparison, we also did molecular docking on the interaction between BMP‐2 and FNIII 12–14 and found 3 hydrogen bonds and 4 salt bridges (Figure 3f,g). We observed hydrogen bond between residue Arg115 in FN and residue Asp30 in chain A of BMP‐2, as well as hydrogen bond between residue Thr116 in FN and residue Asp30 in chain A of BMP‐2. Salt bridges were found between residue Lys142 in FN and residue Asp94 in chain A of BMP‐2, as well as between residue Asp104 in FN and residue Lys101 in chain A of BMP‐2. In addition, hydrogen bond was observed between residue Ser180 in FN and residue Asp53 in chain B of BMP‐2; salt bridges were found between residue Arg230 in FN and residue Glu83 in chain B of BMP‐2, as well as between residue Arg230 in FN and residue Glu119 in chain B of BMP‐2.

In BMP‐2, Asp30 and Glu94 are common binding sites for both the Lys‐Nle peptide and FNIII 12–14. The number of binding sites and the binding energy of the Lys‐Nle peptide are slightly less than that of FN III12‐14, consistent with the SPR results that the binding affinity of Lys‐Nle peptide and FN to BMP‐2 is 36× 10^−9^ and 5× 10^−9^ m, respectively.^[^ ^9^ ^]^ Thus, the results are reliable and showed that both the Lys‐Nle peptide and FNIII 12–14 bind to the “finger” region of one disulfide‐linked BMP‐2 dimer, interacting with both chain A and chain B of BMP‐2.

### 2.4 Immobilization of Copolymer Lys_0.4_Nle_0.6_ onto the 3D Scaffold

**Figure 4:**
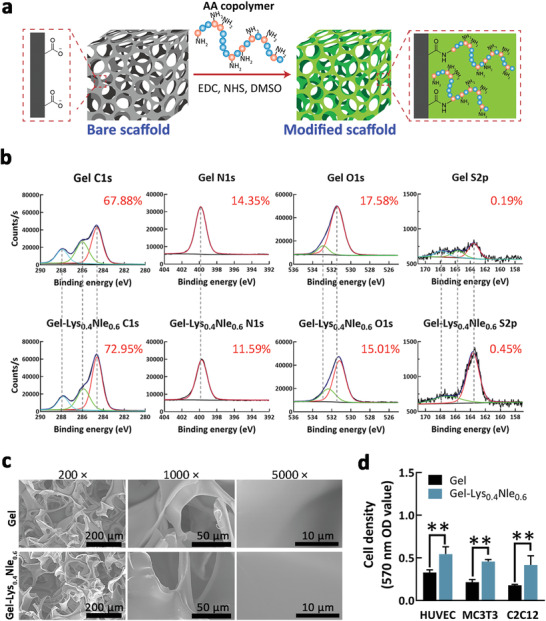
Fabrication and characterization of AA copolymers‐immobilized 3D scaffolds. a) Covalently immobilize the selected AA copolymer to gelatin scaffold (Gel) via a one‐step coupling between the amine groups in Lys_0.4_Nle_0.6_ on the carboxyl groups on the surface of Gel. b) High‐resolution X‐ray photoelectron spectroscopy characterization on modified scaffolds revealed the successful immobilization of Lys_0.4_Nle_0.6_ onto the Gel scaffolds. Black line represents experimental spectrum; blue line represents fitting spectrum; red/green/azure line represents peak decomposition. c) Scanning electron microscope characterization on the morphology of Gel and Gel‐Lys_0.4_Nle_0.6_ scaffolds. d) Cell density in bare Gel and Gel‐Lys_0.4_Nle_0.6_ scaffolds (**p < 0.01, *n* = 3). Representative images are shown. Mean ± SD are shown.

Due to the short in vivo half‐life time, GFs were usually loaded into scaffolds applying for tissue regeneration to protect GFs from protease degradation and prolong the bioactivity of GFs via sustaining release from the scaffolds. We chose the widely used and commercially available gelatin sponge (Gel) as a model 3D scaffold and then covalently tethered the aforementioned optimal AA copolymer, Lys_0.4_Nle_0.6_, to the scaffold surface by coupling the amino groups within copolymers to the carboxyl groups in Gel scaffolds (Figure 4a). The successful preparation of Lys_0.4_Nle_0.6_‐modified 3D Gel scaffold (Gel‐Lys_0.4_Nle_0.6_) was confirmed by the XPS characterization, which indicates the continuously increasing element content of C (from 67.88% to 72.95% owning to Nle) and S (from 0.19% to 0.45% owning to terminal thiol group) (Figure 4b). This conclusion was also supported by observing a decreased concentration of Lys_0.4_Nle_0.6_ after Lys_0.4_Nle_0.6_ copolymer immobilization to the scaffolds (Figure S39, Supporting Information). The results showed that ≈1 mg of polymer was successfully grafted onto the Gel scaffolds (5 mm × 5 mm × 5 mm). A further scanning electron microscope characterization indicated no change of surface morphology on the Gel scaffold before and after Lys_0.4_Nle_0.6_ immobilization (Figure 4c). We evaluated the biocompatibility of Gel‐Lys_0.4_Nle_0.6_ using C2C12 cell, MC3T3 cell, and human umbilical vein endothelial cell (HUVEC) as cell models involved in osteogenesis and angiogenesis, and found that cell density in Gel‐Lys_0.4_Nle_0.6_ was over two times higher than the bare 3D Gel scaffold for all three types of cells (Figure 4d).

### 2.5 Release Behavior of BMP‐2 from 3D Gel‐Lys_0.4_Nle_0.6_ Scaffold

BMP‐2, a FN III12‐14 binding GF, was used as a model in this study for proof‐of‐concept demonstration because BMP‐2 is a FDA‐approved GF that has been extensively used in clinical practice and research to stimulate osteoinduction and bone regeneration. However, BMP‐2 has short in vivo half‐lifetime at about 15 min in spite of the long period for bone regeneration and repair, which leads to dramatically increased dose of BMP‐2 and accompanying risk of serious side effects and heavy economic burden for patients.^[^ ^20^ ^]^

**Figure 5:**
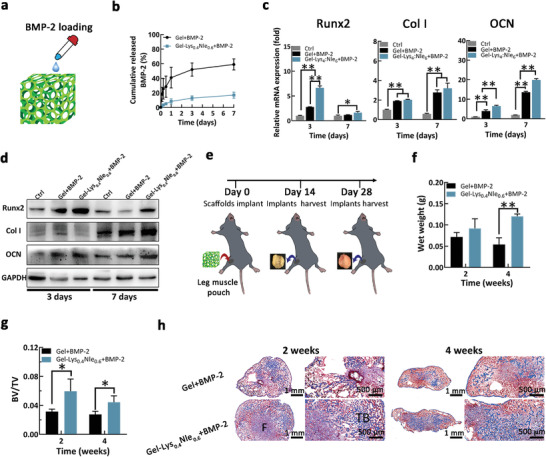
Delivering BMP‐2 within AA copolymers‐immobilized 3D scaffolds induces the osteogenic differentiation in vitro and in vivo. a) BMP‐2 loaded Gel scaffolds. b) Release behavior of BMP‐2 from Gel scaffolds were quantified by ELISA. c) Expression of osteogenic genes marker (corresponding to runt‐related transcription factor 2 (Runx2), type I collagen (Col I), and osteocalcin (OCN)) within C2C12 cells cultured in variable scaffolds for 3 days and 7 days, using cells cultured on the tissue culture plate as the control. d) Western blot analysis on protein expression of Runx2, Col I and OCN within C2C12 cells cultured in variable scaffolds for 3 days and 7 days, using cells cultured on the tissue culture plate as the control. e) The scheme for ectopic bone formation experiments. f) Harvested implants after 2 and 4 weeks were evaluated by bone wet weight. g) 3D SRµCT of obtained ectopic bones and corresponding quantified data of bone volume fraction after implants were harvested 2 and 4 weeks post implantation. h) Histological evaluation of harvested ectopic bone sections by Masson's trichrome staining (F: fibrous tissue, TB: trabecular bone) (*p < 0.05, **p < 0.01, *n* = 3). Representative images are shown. Mean ± SD are shown.

The 3D Gel scaffolds modified with Lys_0.4_Nle_0.6_ were loaded with BMP‐2 by adsorbing BMP‐2 solution into the Gel scaffolds and lyophilizing the scaffolds (Figure 5a). We studied BMP‐2 release in phosphate buffer saline (PBS),^[^ ^21^ ^]^ and found that bare Gel scaffolds had a burst release of BMP‐2 from the very beginning and released over 40% of the loading after 1 day, which is consistent to precedent reports that the widely used gelatin scaffolds have only weak binding to growth factors and have remarkable burst release as the long‐lasting shortcomings.^[^ ^22^ ^]^ In sharp contrast, Gel‐Lys_0.4_Nle_0.6_ scaffolds had sustained release of BMP‐2 from the starting point and released only 17% of the loading after 7 days (Figure 5b). The overcoming of burst release and the sustained release of BMP‐2 from Gel‐Lys_0.4_Nle_0.6_ scaffolds echoed the BMP‐2 binding function of Lys_0.4_Nle_0.6_. A precedent study reported that fibronectin binding domain‐functionalized matrices can significantly delay the release of soluble growth factor PDGF‐BB compared to the matrix functionalized with the fragment that cannot bind the growth factor.^[^ ^14^ ^]^ It is found that 80% of the PDGF‐BB remained within the fibronectin binding domain‐functionalized matrices after 5 days. This implies that the performance of our fibronectin‐mimicking copolymer in the 3D gels is similar to that of the fibronectin binding domain.

### 2.6 Influence of FN‐Mimicking Lys_0.4_Nle_0.6_ Copolymer on the In Vitro Activity of BMP‐2

We continued to investigate the effect of 3D Gel‐Lys_0.4_Nle_0.6_ scaffolds on BMP‐2‐induced osteogenic differentiation of C2C12 cells in vitro, using tissue culture plate and bare 3D Gel scaffolds in the presence of BMP‐2 as controls. Real‐time quantitative polymerase chain reaction (RT‐qPCR) analysis was employed^[^ ^23^ ^]^ and indicated that cells within Gel‐Lys_0.4_Nle_0.6_ scaffolds expressed higher levels of osteogenic differentiation gene markers than both controls, regarding runt‐related transcription factor 2 (Runx2), type I collagen (Col I), and osteocalcin (OCN) (Figure 5c). Further western blot assays showed that cells within Gel‐Lys_0.4_Nle_0.6_ scaffolds also expressed higher protein levels of these markers (Runx2, Col I, and OCN) than both controls, which echoed the mRNA analysis (Figure 5d). The higher gene and protein level of Runx2, Col I, and OCN, which are the downstream of BMP receptor signaling, indicated that the bound BMP‐2 is still active and can activate the BMP receptors. Our experimental result is consistent to the conclusion in precedent literatures that growth factor immobilized on the surface could active corresponding receptors on the cell membranes and maintain the bioactivity.^[^ ^24^ ^]^

### 2.7 In Vivo Ectopic Bone Formation of BMP‐2 Loaded 3D Gel‐Lys_0.4_Nle_0.6_ Scaffolds

We evaluated the in vivo activity of BMP‐2 on the ectopic bone formation in mice by implanting 5 µg BMP‐2 loaded 3D Gel scaffolds, with and without Lys_0.4_Nle_0.6_ modification, into muscle pouches of mice (Figure 5e). We chose the model of ectopic bone formation rather than bone healing, because the ectopic bone formation model utilizes of uniquely isolated in vivo ectopic implant sites to study the effects on bioactivity of exogenous BMP‐2.^[^ ^25^ ^]^ The bone healing model, however, has complicated in vivo environment and the presence of endogenous BMP‐2, which will make the outcome difficult to have simple correlation with exogenous BMP‐2. In particular, a low dose (5 µg) of BMP‐2 was selected to evaluate the ability of the fibronectin mimicking copolymer (Lys_0.4_Nle_0.6_) in enhancing the in vivo activity of BMP‐2.

After 2 and 4 weeks post‐operation, we harvested the implants and found the formation of bone in both groups. It is noteworthy that bones formed in the Gel‐Lys_0.4_Nle_0.6_+BMP‐2 group were superior to that formed in the Gel+BMP‐2 group, regarding the bone volume and wet weight (Figure 5f). Micro‐CT examination showed a substantially higher ratio of bone volume/total volume in the Gel‐Lys_0.4_Nle_0.6_+BMP‐2 group than that in the Gel+BMP‐2 group after 2 and 4 weeks post‐operation (Figure 5g). Using Masson's trichrome staining, histological analysis showed that the formed ectopic bones were filled with fibrous tissue, a few bone marrows, and some trabecular bone tissues after 2 weeks in the Gel+BMP‐2 group (Figure 5h). After 4 weeks, we observed that trabecular bones were surrounded with rich medulla ossium rubra and lipocytes. Moreover, the content of trabecular bone in the Gel‐Lys_0.4_Nle_0.6_+BMP‐2 group was significantly higher than that in the Gel+BMP‐2 group. These results showed that modifying the BMP‐2 binding copolymer Lys_0.4_Nle_0.6_ to 3D Gel scaffolds could promote the in vivo activity of BMP‐2 substantially, which supported our design of amphiphilic copolymers in mimicking FN III12‐14, the GF binding domain of fibronectin.

## 3 Conclusion

We design and synthesize a library of synthetic mimics of FN III12‐14, the growth factor (GF) binding domain of fibronectin, using positively charged amphiphilic AA copolymers. Lys_0.4_Nle_0.6_ is identified as the optimal copolymer that binds directly to a large variety of GFs from different GF families with high binding affinity. Lys_0.4_Nle_0.6_ is easily modified to 3D Gel scaffolds via covalent bonding to give the functional 3D Gel‐Lys_0.4_Nle_0.6_ scaffolds that addresses the burst release of BMP‐2, a long‐lasting and formidable challenge in using BMP‐2, and substantially enhances the in vivo activity of BMP‐2 for bone formation. All these proof‐of‐concept demonstrations support our hypothesis in mimicking the GF binding domain of fibronectin using synthetic polymers. Even though protein mimics have been studied using synthetic peptides,^[^ ^26^ ^]^ our strategy opens new avenues in designing GF binding polymers and preparing advanced GF delivery systems. Moreover, the structure diversity, excellent in vivo stability, low cost, and easy preparation in large quantity for AA copolymers reveal their great potential in practical application.

## 4 Experimental Section

### Synthesis of AA Copolymers

AA copolymers were prepared from the ring‐opening polymerization (ROP) on NCAs initiated by a primary amine, 2‐(tritylthio)ethanamine.^[^ ^27^ ^]^ A representative synthesis of the heterochiral random copolymer poly(d,l‐lysine)_0.4_‐r‐poly(d,l‐Norleucine)_0.6_ (Lys_0.4_Nle_0.6_) was described below, and all the other copolymers were synthesized using a similar route. In a nitrogen‐purged glovebox, anhydrous tetrahydrofuran (THF) solution of N*ε*‐*tert*‐butyloxycarbonyl‐d,l‐Lys NCA (Boc‐d,l‐Lys NCA) (2 mL, 0.1 m), and d,l‐Nle‐NCA (3 mL, 0.1 m) were mixed in a dried reaction vial, followed by adding a THF solution of 2‐(tritylthio)ethanamine (0.25 mL, 0.1 m) to the reaction. The mixture was stirred at room temperature for 3 days under a N_2_ atmosphere. Afterward, the reaction mixture was removed from the glovebox and poured into cold petroleum ether (40 mL) to precipitate out a white fluffy solid, which was then collected by centrifugation and dried under airflow. The collected solid was dissolved in THF (1.5 mL), and then petroleum ether (40 mL) was added to precipitate out the polymer again. This dissolution‐precipitation process was repeated two more times to obtain NHBoc protected AA copolymer poly(Boc‐d,l‐Lys)_0.4_‐r‐poly(d,l‐Nle)_0.6_. Boc and trityl protecting groups were removed from the side chain of amines and the terminal thiol, respectively, by treating the polymer with trifluoroacetic acid (TFA) containing 5% (v/v) under gentle shaking at room temperature (rt) overnight. The resulting viscous liquid was dissolved in MeOH (0.5 mL), and cold Et_2_O (40 mL) was added to the mixture to precipitate out a solid. This dissolution‐precipitation process was repeated two more times to give a white solid, which was then dissolved in milli‐Q and lyophilized to obtain the final product of AA copolymer Lys_0.4_Nle_0.6_ in the form of TFA salt (67 mg, 80% yield).

### Gel Permeation Chromatography (GPC) Analysis

AA copolymers at the NHBoc protected stage were characterized by GPC using *N,N*‐dimethyl formamide (DMF), supplemented with 0.01 m LiBr, as the mobile phase at a flow rate of 1 mL min^−1^ at 50 °C. Sample solutions were filtered through 0.22 µm polytetrafluoroethylene filters before GPC analysis. The relative number average molecular weight (*M_n_*) and dispersity index (*Đ*) were calculated from a calibration curve using poly(methylmethacrylate) PMMA as internal standards on a Breeze 2 software. Degree of polymerization (DP) was determined from the obtained *M_n_* in GPC analysis and ratio of two subunits within the AA copolymer chain.

### Screening on GF Binding to AA Copolymers

AA copolymers were tethered to glass surfaces via polymer terminal thiol group using OEG_8_ as an antifouling layer below the copolymer chains by following the protocol in our recent publication.^[^ ^19^ ^]^ Briefly, glass slides were cleaned under ultraviolet (UV) irradiation for 25 min and then immersed in 2% (v/v) of (3‐aminopropyl)triethoxysilane in anhydrous toluene at rt overnight. After an annealing process at 80 °C for 2 h, the glass slides were covered with 50‐well chambered coverslips, and 10 µL of maleimide‐octaethylene glycol‐*N*‐hydroxysuccinimide at 10 mg mL^−1^ in PBS (pH 7.4) were added into each well. After incubation for 3 h, the antifouling OEG_8_ layer was successfully modified to the glass surface. An aliquot of 10 µL AA copolymers or RGD peptide at 2 mg mL^−1^ was added to each well, and the glass slides were incubated at rt overnight in a humidifying chamber. The glass slides were washed using ethanol and Milli‐Q water repeatedly and then blocked with 100 × 10^−3^ m thioglycerol at rt for 1 h. The bare glass and polymer‐modified glass are characterized by XPS using an Al K*α* source and confirmed the successful modification of polymer to glass surface. The thickness of polymer layer on the glass surface was measured using a spectroscopic ellipsometer.

An aliquot of 10 µL GF (5 µg mL^−1^) was incubated on the aforementioned AA copolymer‐immobilized surfaces for 3 h, and GF binding to copolymers was detected by immunofluorescence assay using a rabbit polyclonal antibody to GFs (1:200) for overnight incubation at 4 °C, followed by incubation with Alexa Fluor 488‐conjugated goat anti‐rabbit IgG H&L (1:200) for 90 min at rt. The fluorescence intensity in each well was acquired on a fluorescent scanner that was equipped with the Imagequant software.

### Surface Plasmon Resonance

The AA copolymer (Lys_0.4_Nle_0.6_) or the Lys‐Nle peptide was immobilized, via its terminal thiol group, onto a CM5 chip to reach a resonance unit of 1951.1 and 1123.9, according to the manufacturer's instructions. GFs or BSA was flown at increasing concentrations in the running buffer (PBS‐T) at 50 µL min^−1^. The sensor chip was regenerated with Gly‐HCl (pH 1.5) for every cycle. Specific bindings of proteins to Lys_0.4_Nle_0.6_ were calculated using a nonfunctionalized channel as the reference. Experimental results were fitted with Langmuir binding kinetics using BIAevaluation software.

### Molecular Dynamics Simulation

The structure of Lys‐Nle peptide was build and optimized by MD simulation. The Lys‐Nle peptide was neutralized by adding sodium/chlorine counter ions and solvated in a cubic box fulling of TIP3P water molecules with 10 Å solvent layers between the box edges and solute surface. All MD simulations were performed using AMBER16.^[^ ^28^ ^]^ The AMBER FF14SB force field was applied and the SHAKE algorithm was used to restrict all covalent bonds involving hydrogen atoms with a time step of 2 fs. The Particle mesh Ewald (PME) method was employed to treat long‐range electrostatic interactions. For each solvated system, two steps of energy minimization were performed before the heating step. The first 4000 cycles of energy minimization were performed with all heavy atoms restrained with 50 kcal (mol^−1^ Å^−2^), whereas solvent molecules and hydrogen atoms were free to move. Then, nonrestrained minimization was carried out involving 2000 cycles of steepest descent energy minimization and 2000 cycles of conjugated gradient energy minimization. Afterward, the whole system was first heated from 0 to 300 K in 50 ps using Langevin dynamics at a constant volume and equilibrated for 400 ps at a constant pressure of 1 atm. A weak constraint of 10 kcal (mol^−1^ Å^−2^) was used to restrain all the heavy atoms during the heating. Periodic boundary dynamics simulations were carried out for the whole system under an NPT (constant composition, pressure, and temperature) ensemble at a constant pressure of 1 atm and 300 K in the production step. In the production phase, a 100 ns simulation was carried out.

### Molecular Docking

Molecular docking was conducted using MOE v2018.0101. The protein–protein docking protocol in MOE was applied for molecular docking. The smaller protein (a smaller number of residues) usually is set as the ligand and the bigger one as the receptor. Here the protein PtoCYCD3 was defined as the ligand and others as the receptor. A multistage method was applied for generating poses and then ranking them. Starting from a coarse‐grained (CG) model to reduce the computational search space, exhaustive sampling was carried out to generate a set of initial poses. The Hopf fibration is used to generate a set of uniformly distributed rotations, and a fast Fourier transform (FFT) is used to sample all translations for a given rotation. Afterward, a minimization process built around a staged convergence protocol was undertaken. The best‐ranked conformation was selected as the final (probable) binding mode. Molecular graphics were generated by PyMOL.

### Fabrication and Characterization of AA Copolymer‐Modified 3D Scaffold

To a solution of 1‐(3‐dimethylaminopropyl)‐3‐ethylcarbodiimide hydrochloride (EDC, 10 mg) and *N*‐hydroxysuccinimide (NHS, 2.5 mg) in dimethyl sulfoxide (DMSO, 1 mL) was added AA copolymer (2 mg), and the resulting solution was used to immerse the Gel scaffolds (5 mm × 5 mm × 5 mm) for copolymer conjugation. After incubation in the above solution at rt for 2 h, the Gel scaffolds were rinsed five times with ethanol and Milli‐Q water repeated to remove nontethered copolymer and residual chemicals from AA copolymer‐modified Gel scaffold. After overnight lyophilization and sterilization under UV for 1 h, Gel scaffolds were used in the following experiments. In preliminary experiments, the Gel scaffold's infiltration capacity was tested and it was found that 45 µL of solution can be absorbed by each scaffold (5 mm × 5 mm × 5 mm). Therefore, 40 µL of BMP‐2 solution was chosen to be loaded into each Gel scaffold to ensure equal loading. The amount of BMP‐2 used per scaffold is 1 µg for cell experiments and 5 µg for animal experiments. The resulting 3D Gel scaffolds were characterized by XPS using an Al K*α* source and confirmed the successful modification of AA copolymer to 3D Gel scaffolds.

### ELISA Assay

To evaluate the release behavior of BMP‐2 from Gel scaffolds, the specimens were immersed in 1 mL PBS (0.01 m, pH 7.4) at 4 °C, and the PBS buffer was exchanged at the predetermined time point of 3 h, 6 h, 12 h, 24 h, 3 days, and 7 days. The BMP‐2 concentration in PBS, as collected at each time point, was detected using a human BMP‐2 ELISA kit by following the manufacturer's instructions.

### Cell Culture

C2C12 cells, MC3T3 cells, and HUVEC were cultured in Petri dishes using DMEM containing 10% (v/v) fetal bovine serum (FBS), 100 U mL^−1^ penicillin, 100 µg mL^−1^ streptomycin, and 1% (v/v) l‐glutamine. The culture medium was changed every two days until 80–90% confluence was achieved. Cells at passage 3–10 were used for in vitro experiments.

### MTT Assay

Cell adhesion was determined by MTT. Briefly, C2C12 cells, MC3T3 cells, and HUVEC were cultured on the AA copolymer‐modified scaffolds in the 24‐well plate (*n* = 3). After 24 h incubation, 100 µL of MTT solution (5 mg mL^−1^) was added to each well, and the formazan crystals were formed after 4 h incubation with MTT. The scaffolds were transferred into a new 24‐well plate to dissolve the formazan within each scaffold in 500 µL DMSO in 10 min. After thorough mixing, 200 µL of each solution was transferred into a 96‐well plate in duplicate. The OD value of the solution in each well was measured at 570 nm on a plate reader.

### Gene Expression Analysis

C2C12 cells was employed in this study because BMP‐2 can convert the differentiation pathway of C2C12 myoblasts into osteoblast lineage. Therefore, it can rule out the effects by other factors in osteogenic differentiation.^[^ ^29^ ^]^ C2C12 cells were cultured on the BMP‐2‐loaded Gel scaffolds for 3 days and 7 days, respectively, and then total RNA was extracted using the TRIZOL reagent and detected by measuring the OD values at 260 nm. Reverse transcription of mRNA into the first‐strand complementary DNA (cDNA) was conducted using the PrimeScript RT reagent Kit. Quantitative polymerase chain reaction (qPCR) analysis was performed to examine the gene expression levels of osteogenic protein markers (Runx2, Col I, OCN) using a real‐time qPCR system containing 10 µL SYBR Premix Ex TaqTM, 0.4 µL of both forward and reverse primers at 50 × 10^−6^ m (listed in Table S3, Supporting Information), 1 µL of cDNA template, and 8.2 µL of RNase free water. The relative expression level was calculated by the Livak method.

### Protein Expression Analysis

Western blot analysis was performed to detect the protein expression of Runx2, Col I, OCN, and GAPDH. C2C12 cells were cultured on the BMP‐2 loaded Gel scaffolds for 3 days and 7 days, respectively, and then cells were lysed using RIPA lysis buffer containing 1 × 10^−3^ m PMSF. The electrophoresis was performed in 8% SDS‐PAGE gel to separate the protein samples. Then, proteins were transferred from the gel onto PVDF membranes, and the membranes were incubated with specific primary antibodies against Runx2 (1:1000, v/v), Col I (1:1000, v/v), OCN (1:1000, v/v), and GAPDH (1:1000, v/v) overnight. These samples were incubated with HRP‐linked secondary antibodies (1:1000, v/v) against the primary antibodies for 90 min. Then HRP signals were detected via the ECL chemiluminescence reagent by Image Quant LAS 4000, and the chemiluminescent intensity was calculated using the Imagequant software.

### Ectopic Bone Formation In Vivo

The ectopic bone formation experiment was performed as described below. Twelve male C57BL/6 mice at ≈20 g were randomly allocated into 2 groups (*n* = 6 for each group). Implants were prepared by loading 5 µg of BMP‐2 into the scaffolds with or without Lys_0.4_Nle_0.6_ modification and then were lyophilized overnight and stored at −20 °C until use. Implants were placed in the leg muscle pouches of the mice. All animal‐related procedures were approved by the Animal Care and Use Committee of Shanghai General Hospital. After 2 and 4 weeks postoperation, mice were sacrificed by an intraperitoneal overdose injection of pentobarbital sodium solution and implants were harvested. The implants were imaged by a digital camera and measured for wet weight. Synchrotron radiation‐based microcomputed tomography (SRµCT) was measured at beamline BL13W of Shanghai Synchrotron Radiation Facility (SSRF) at a voxel size of 6.5 µm. After being decalcified in 10% EDTA, the samples were sectioned and evaluated using Masson's trichrome staining.

### Statistical Analysis

All data were presented as the mean ± SD. For comparisons between two groups, we used unpaired *t*‐tests. For comparisons among multiple groups, one‐way ANOVA was used. Moreover, *p*‐value < 0.05 was considered to be statistically significant. **: significantly different with the *p*‐value < 0.01. *: significantly different with the *p*‐value < 0.05. ns means no significance. Quantitative data plotting and statistical calculations were performed in GraphPad Prism version 8.0 software.

## Conflict of Interest

R.L. and W.Z. are co‐inventors on a patent application covering reported materials and application to bind growth factors. All remaining authors declare no competing interests.

## Supporting information

Supporting Information

## Data Availability

The data that support the findings of this study are available from the corresponding author upon reasonable request.
