# Supplementary material for: Statistic Copolymers Working as Growth Factor‐Binding Mimics of Fibronectin
Source: Adv Sci (Weinh). 2022 May 15;9(21):2200775. doi: 10.1002/advs.202200775 (PMC9313494; doi:10.1002/advs.202200775)
Supplement: Supplementary file 1 — Supporting Information [file ADVS-9-2200775-s001.pdf]

ADVANCED SCIENCE
Open Access

Supporting Information

for *Adv. Sci.*, DOI 10.1002/advs.202200775

Statistic Copolymers Working as Growth Factor-Binding Mimics of Fibronectin

*Wenjing Zhang, Yueming Wu, Qi Chen, Haodong Zhang, Min Zhou, Kang Chen, Chuntao Cao, Han Guo, Jianrong Xu, Honglai Liu, Haodong Lin, Changsheng Liu and Runhui Liu**

Supporting Information

Statistic Copolymers Working as Growth Factor-Binding Mimics of Fibronectin

*Wenjing Zhang, Yueming Wu, Qi Chen, Haodong Zhang, Min Zhou, Kang Chen, Chuntao Cao, Han Guo, Jianrong Xu, Honglai Liu, Haodong Lin, Changsheng Liu and Runhui Liu**

W. Zhang, M. Zhou, R. Liu
State Key Laboratory of Bioreactor Engineering
East China University of Science and Technology
Shanghai 200237, China
Email: rliu@ecust.edu.cn
Y. Wu, Q. Chen, H. Zhang, K. Chen, C. Cao, C. Liu, R. Liu
Key Laboratory for Ultrafine Materials of Ministry of Education
Frontiers Science Center for Materiobiology and Dynamic Chemistry
Research Center for Biomedical Materials of Ministry of Education
School of Materials Science and Engineering
East China University of Science and Technology
Shanghai 200237, China
H. Guo
Shanghai Synchrotron Radiation Facility (SSRF)
Shanghai Advanced Research Institute
Chinese Academy of Sciences
Shanghai 201204, China
J. Xu
Academy of Integrative Medicine
Shanghai University of Traditional Chinese Medicine
Shanghai 201203, China.
H. Liu
School of Chemistry and Molecular Engineering
East China University of Science and Technology
Shanghai 200237, China.
H. Lin

Department of Orthopedic Surgery
Shanghai General Hospital
Shanghai Jiao Tong University School of Medicine
Shanghai 200080, China.

1. Materials and Methods

1.1. General information and Materials

Anhydrous tetrahydrofuran (THF) (109731) was purchased from Sigma-Aldrich and used for polymerization. The rest chemical reagents and solvents were purchased from Adamas-beta and used without further purification. Sulfydryl assay kit was purchased from Solarbio (BC1453). Glass slides (25 × 76 mm, HM-0376) and glutaraldehyde (14376B) were obtained from Taitan. (3-aminopropyl)triethoxysilane (01197015) was purchased from Adamas-beta. 50-well silicone coverslips (103350) were purchased from Grace Biolabs. Maleimide-octaethylene glycol-*N*-hydroxysuccinimide were purchased from Biomatrik Inc. RGDSPC peptide and custom-synthesized Lys-Nle peptide were obtained from Synpeptide. Thioglycerol was obtained from Bailingwei Technology. Synthesized intermediates were purified using a SepaBean machine equipped with Sepaflash columns produced by Santai Technologies Inc. in China. Recombinant human bone morphogenetic protein-2 (BMP-2) was purchased from Shanghai Rebone Biomater. Recombinant human fibroblast growth factor-10 (FGF-10; CR11), FGF-21(C223), hepatocyte growth factor (HGF; CJ72), epidermal growth factor (EGF; C029), platelet derived growth factor-AA (PDGF-AA; CH79), PDGF-BB (C199), transforming growth factor-β1 (TGF-β1; CA59) were purchased from Novoprotein (Non-glycosylated proteins produced in *Escherichia coli*). C2C12 cells, MC3T3 cells and human umbilical vein endothelial cells (HUVEC) were purchased from the American Type Culture Collection (ATCC). Absorbable gelatin sponges (Gel; 20163642299) were purchased from Xiang'en Med. Mouse anti-runt-related transcription factor 2 (Runx2; sc390715) was obtained from Santa Curz. Rabbit anti-BMP-2 (ab14933), rabbit anti-type I collagen (Col I; ab260043), rabbit anti-osteocalcin (OCN; ab93876), rabbit anti-glyceraldehyde-3-phosphate dehydrogenase (GAPDH; ab9485) and Alexa Fluor® 488-conjugated goat anti-rabbit IgG H&L (ab150077) were purchased from Abcam. Horse radish peroxidase (HRP)-linked anti-rabbit IgG (7074) and HRP-linked anti-mouse IgG (7076) were obtained from Cell Signaling Technology. Rabbit anti-PDGFA (A17434), rabbit anti-PDGFB (A1195), rabbit anti-FGF-10 (A1201), rabbit anti-FGF-21 (A3908), rabbit anti-TGF-β1 (A15103), rabbit anti-HGF (A1193) were purchased from Abclonal. Rabbit anti-EGF antibody was purchased from Bioss

(bs-4567R). Human BMP-2 enzyme-linked immunosorbent assay (ELISA) kit (EHC172) was purchased from Neobioscience Technology. Fetal bovine serum (FBS; 10099141), penicillin-streptomycin (15140163), L-Glutamine (25030081), fluorescein isothiocyanate (FITC)-phalloidin (F432) and ECL chemiluminescence reagent (34577) were obtained from Thermo Fisher Scientific. RNase free water (R0021), RIPA lysis buffer (P0013B), phenylmethanesulfonyl fluoride (PMSF; ST506) and sodium dodecyl sulfate-polyacrylamide gel (SDS-PAGE) kit (P0012AC) were purchased from Beyotime Biotech. Methyl thiazolyl tetrazolium (MTT; M2128) and 2-(4-Amidinophenyl)-6-indolecarbamidine dihydrochloride (DAPI; D9542) and fluorescamine (F9015) were purchased from Sigma-Aldrich. TRIZOL reagent (9190), PrimeScript RT reagent Kit (RR037A), and SYBR Premix Ex TaqTM (RR420A) were purchased from Takara. Forward and reverse primers were purchased form Sangon Biotech. Polyvinylidene difluoride (PVDF) membranes (IPVH00010) were obtained from Millipore. Dulbecco's modified Eagle's medium (DMEM; SH30243.01), phosphate buffer saline (PBS; SH30256.01), CM5 sensor chips (BR100012) and thiol-coupling kit (BR100557) were purchased from GE Healthcare.

Gel permeation chromatography (GPC) characterization was performed on a Waters GPC instrument equipped with a Waters 1515 isocratic HPLC pump and a Waters 2414 refractive index detector. The GPC were equipped with a Tosoh TSKgel Alpha-2500 column (particle size 7 μm) and a Tosoh TSKgel Alpha-3000 column (particle size 7 μm) linked in series. The X-ray photoelectron spectroscopy (XPS) spectra were recorded on X-ray photoelectron spectrophotometer (ESCALAB 250Xi, Thermo Fisher) using an Al Kα source with a quartz-crystal monochromator. The surface morphology of Gel scaffolds was observed on a scanning electron microscope (JSM-6360LV, JEOL). Optical density (OD) values in MTT assay and ELISA assay were recorded on a plate reader (SPECTRAmax 384, Molecular Devices). Immunofluorescence photographs on screening GFs binding to AA copolymers and chemiluminescence photographs on western blot analysis were captured on Image Quant LAS 4000 (GE Healthcare) and the intensity was quantified using the Imagequant software (GE Healthcare). Surface plasmon resonance (SPR) measurements were made with a Biacore T200 SPR system (GE Healthcare). Micrographs of cell migration were captured on a confocal laser canning microscopy (Nikon A1R). Quantification of gene expression, corresponding to common osteogenic differentiation markers, was performed using real-time quantitative reserve transcription polymerase chain reaction system (Bio-Rad CFX96). Nuclear magnetic resonance (NMR) characterizations were performed on a Bruker spectrometer at 400 MHz. ^{1}H NMR chemical shifts were referenced to the resonance for

residual protonated solvent (δ 4.79 for D_2O, δ 2.50 for DMSO-*d*6). The thickness of polymer layer on the glass surface was measured using a spectroscopic ellipsometer (J. A. Woollam RC2 UI).

1.2. Fluorescamine protein assay

The amount of surface tethered amino acid copolymer within the 3D Gel scaffold was analyzed by fluorescamine protein assay. The samples were collected before and after the interaction between Gel scaffolds and the amino acid copolymer. The scaffolds were washed five times with ethanol and Milli-Q water repeatedly and the buffer solutions were also collected. The samples were incubated with fluorescamine at 1 mM concentration for 10 min before reading fluorescence intensity on the plate reader.

1.3 Sulfydryl assay

The determination of thiol groups in the copolymers was measured according to the guideline of the sulfydryl assay kit. The concentration of 2-nitrothiobenzoate was used to calculate the number of reactive sulfydryl groups. The mixture of polymers and 2-nitrothiobenzoate was transferred into a 96-well plate in duplicate. The OD value of the solution in each well was measured at 412 nm on a plate reader. This result shows that 2 mg/mL of copolymer contains 0.166 μM of thiol group.

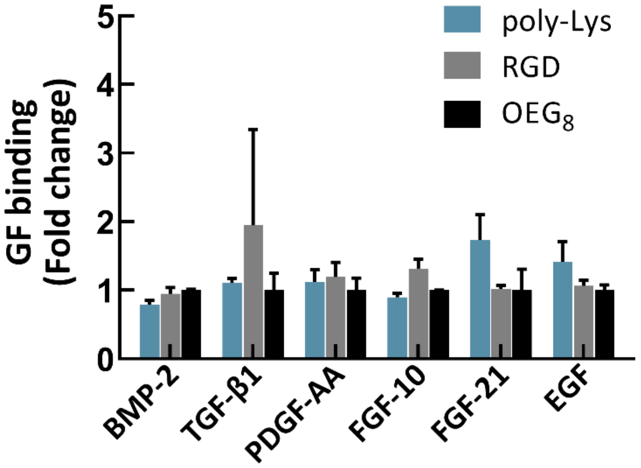

Figure S1. GF binding ability to homopolymer poly-Lys as evaluated from GF adsorption study.

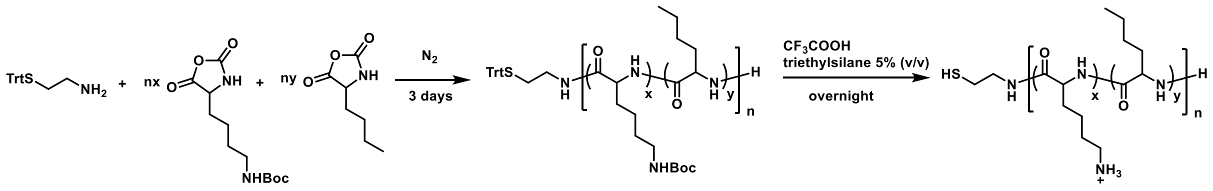

Scheme S1. Synthesis of thiol-terminated Lys_xNle_y ($x+y=1$, $y=0.1$-0.7, $n=30$).

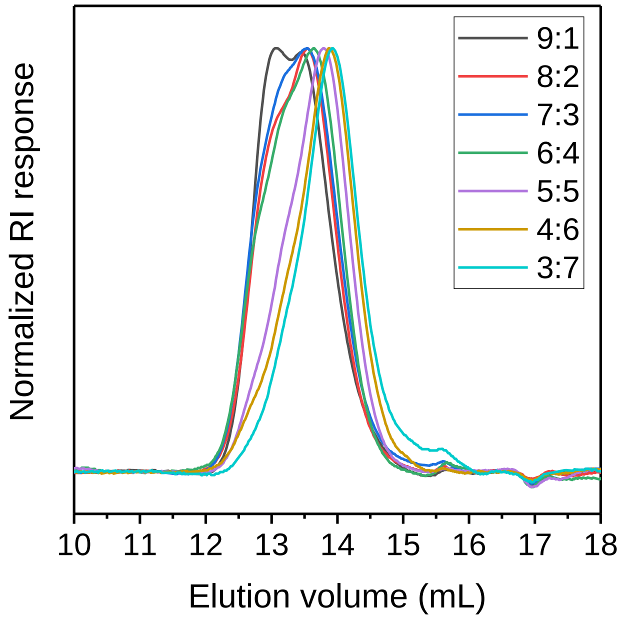

Figure S2. GPC traces of thiol-terminated Lys_xNle_y at the sidechain and N-terminal protected stage using DMF supplemented with 0.01 M LiBr as the mobile phase.

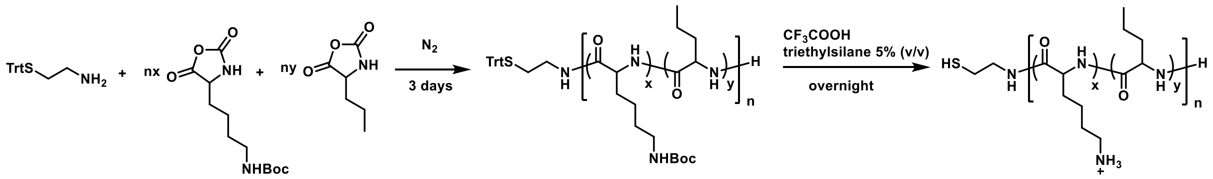

Scheme S2. Synthesis of thiol-terminated Lys_xNva_y (x+y=1, y=0.1-0.7, n=30).

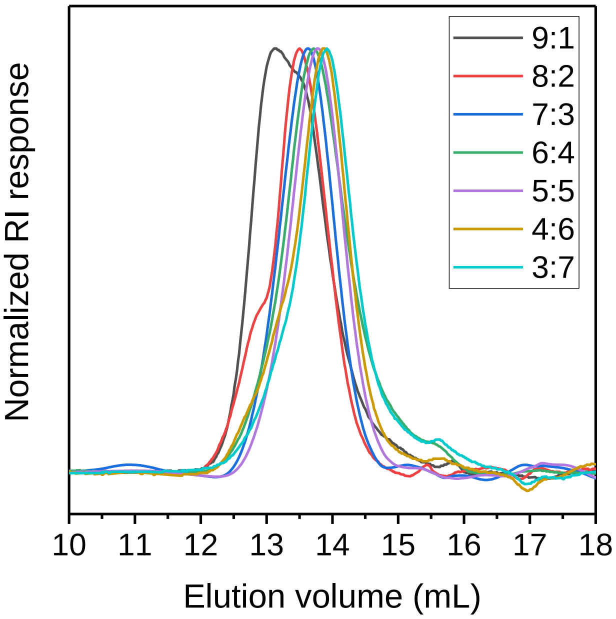

Figure S3. GPC traces of thiol-terminated Lys_xNva_y at the sidechain and N-terminal protected stage using DMF supplemented with 0.01 M LiBr as the mobile phase.

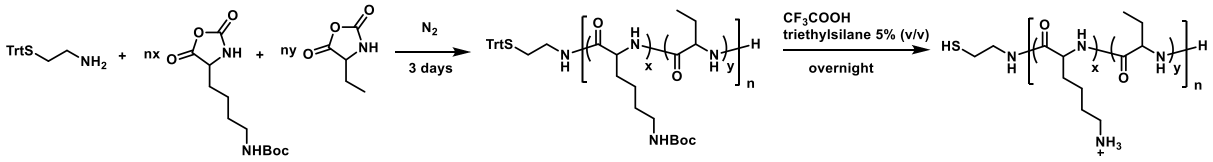

Scheme S3. Synthesis of thiol-terminated Lys_xAba_y (x+y=1, y=0.1-0.7, n=30).

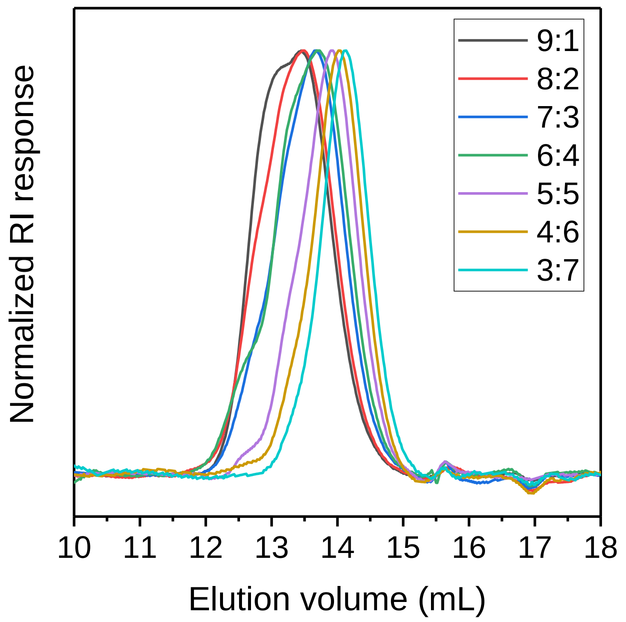

Figure S4. GPC traces of thiol-terminated Lys_xAba_y at the sidechain and N-terminal protected stage using DMF supplemented with 0.01 M LiBr as the mobile phase.

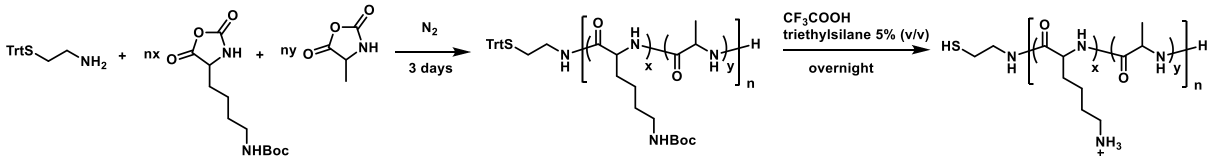

Scheme S4. Synthesis of thiol-terminated Lys_xAla_y (x+y=1, y=0.1-0.7, n=30).

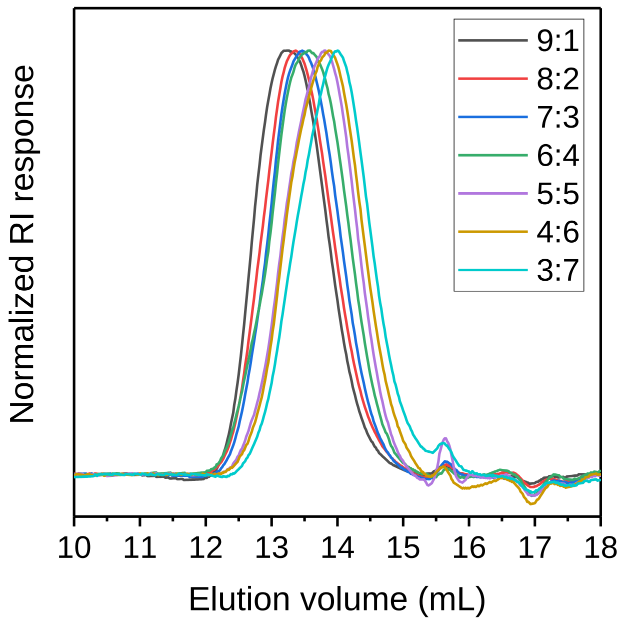

Figure S5. GPC traces of thiol-terminated Lys_xAla_y at the sidechain and N-terminal protected stage using DMF supplemented with 0.01 M LiBr as the mobile phase.

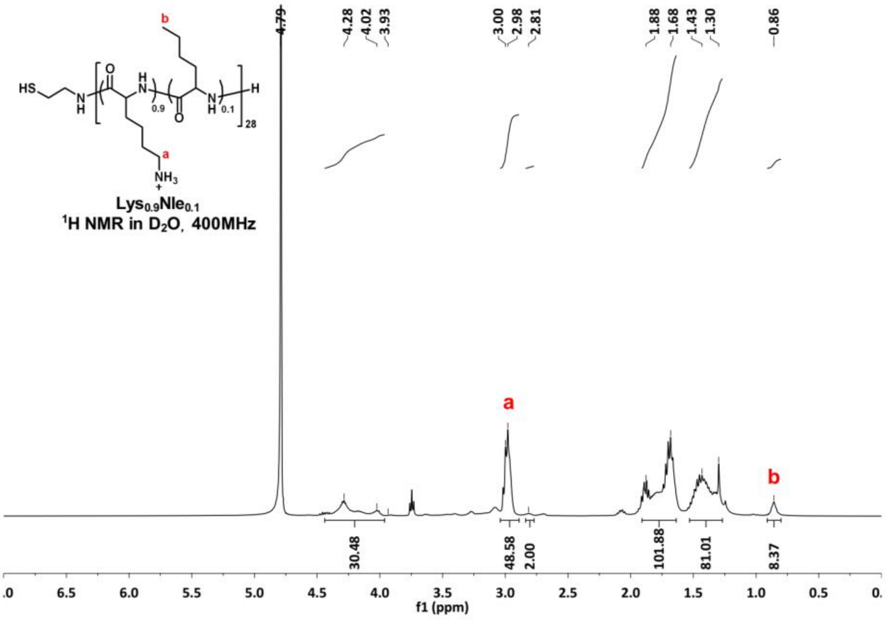

Figure S6. ^{1}H NMR spectrum of $Lys_{0.9}Nle_{0.1}$ in D_2O, 400 MHz.

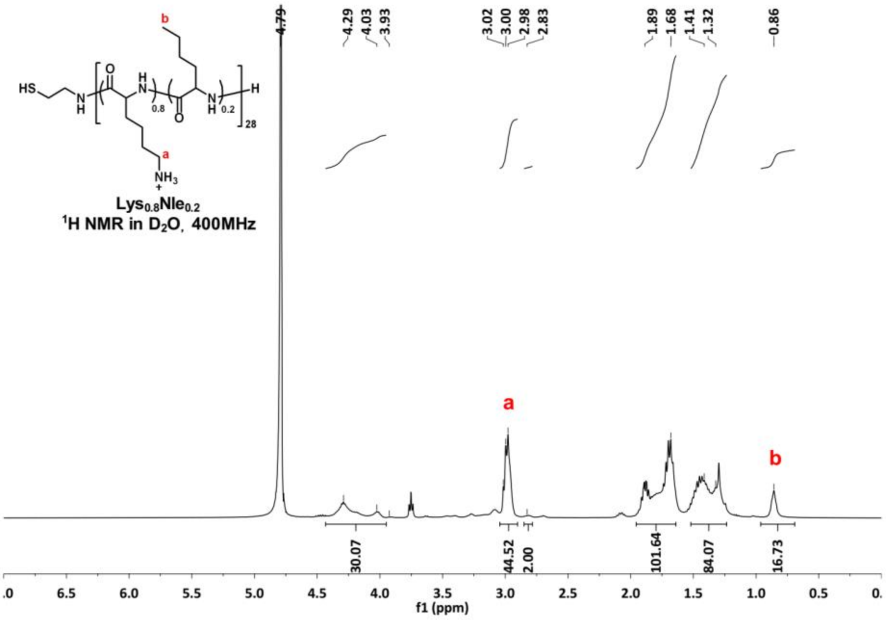

Figure S7. ^{1}H NMR spectrum of $Lys_{0.8}Nle_{0.2}$ in D_2O, 400 MHz.

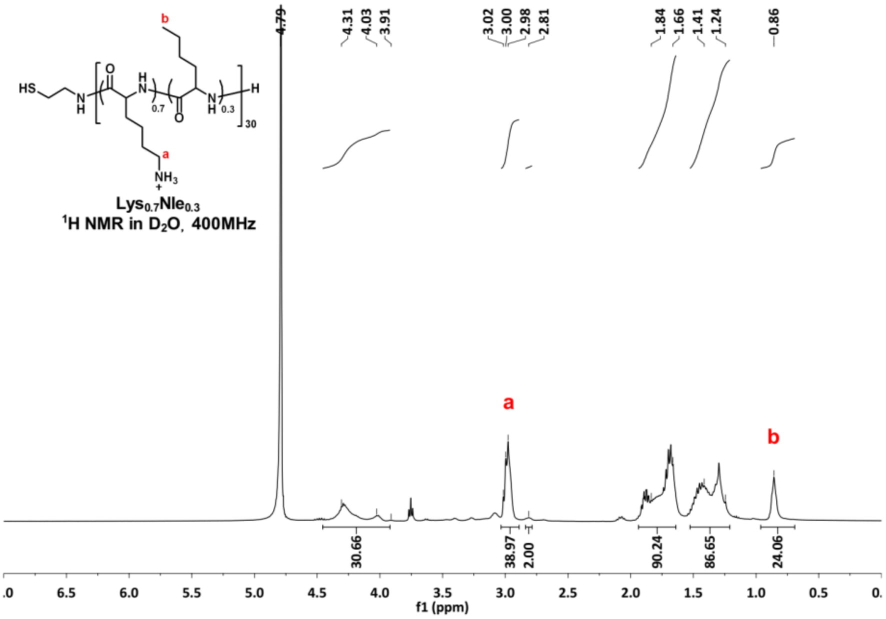

Figure S8. ^{1}H NMR spectrum of $Lys_{0.7}Nle_{0.3}$ in D_2O, 400 MHz.

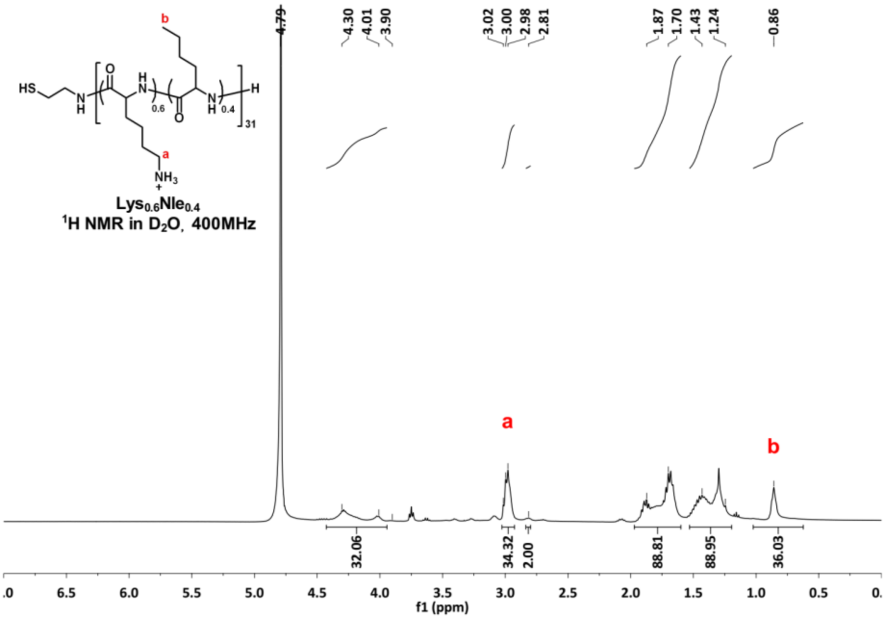

Figure S9. ^{1}H NMR spectrum of $Lys_{0.6}Nle_{0.4}$ in D_2O, 400 MHz.

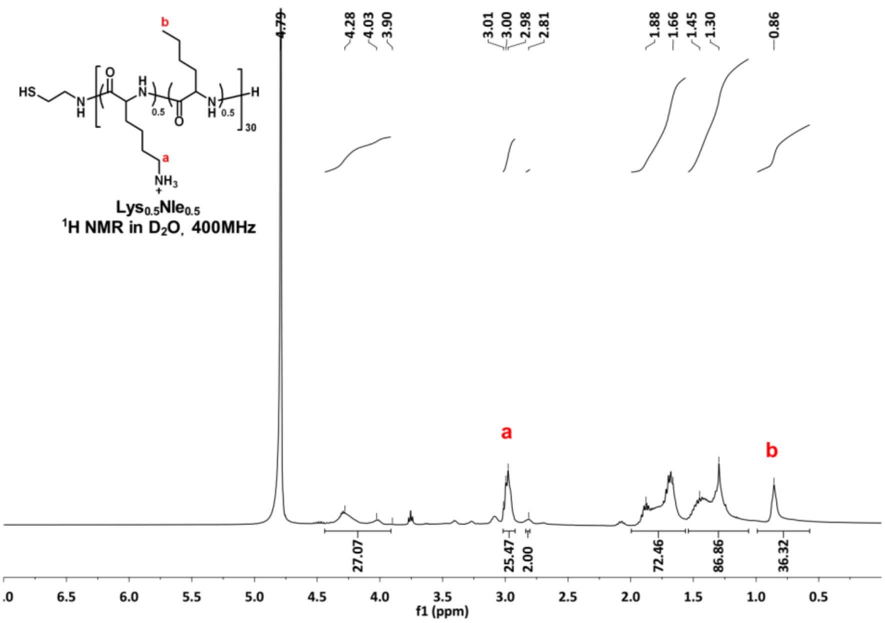

Figure S10. ^{1}H NMR spectrum of $Lys_{0.5}Nle_{0.5}$ in D_2O, 400 MHz.

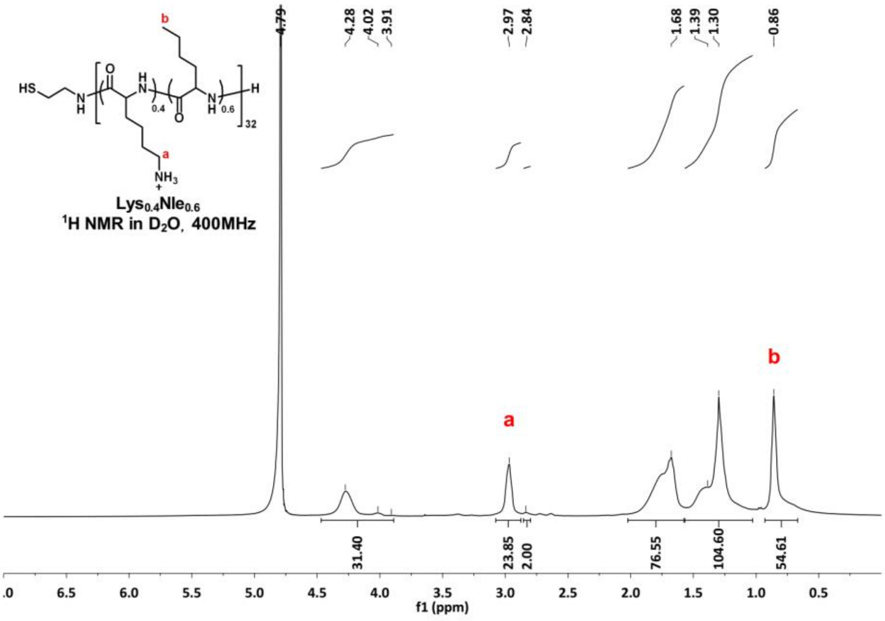

Figure S11. ^{1}H NMR spectrum of $Lys_{0.4}Nle_{0.6}$ in D_2O, 400 MHz**.**

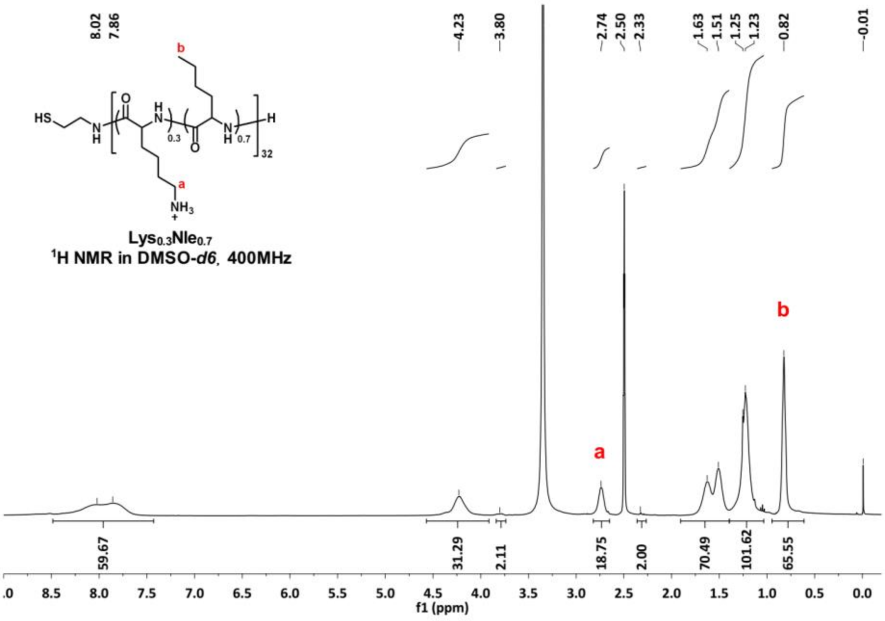

Figure S12. ^{1}H NMR spectrum of $Lys_{0.3}Nle_{0.7}$ in DMSO-*d*6, 400 MHz.

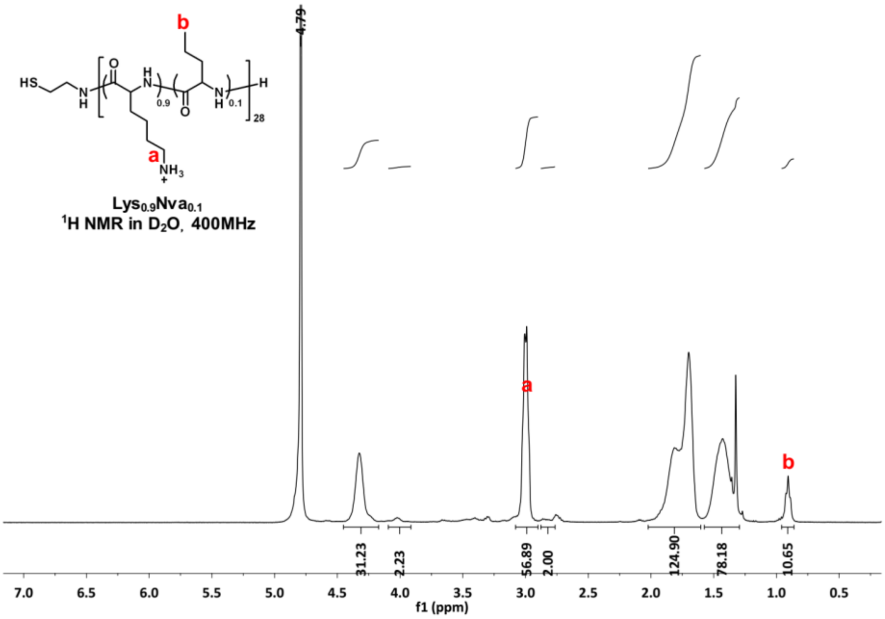

Figure S13. ^{1}H NMR spectrum of $Lys_{0.9}Nva_{0.1}$ in D_2O, 400 MHz.

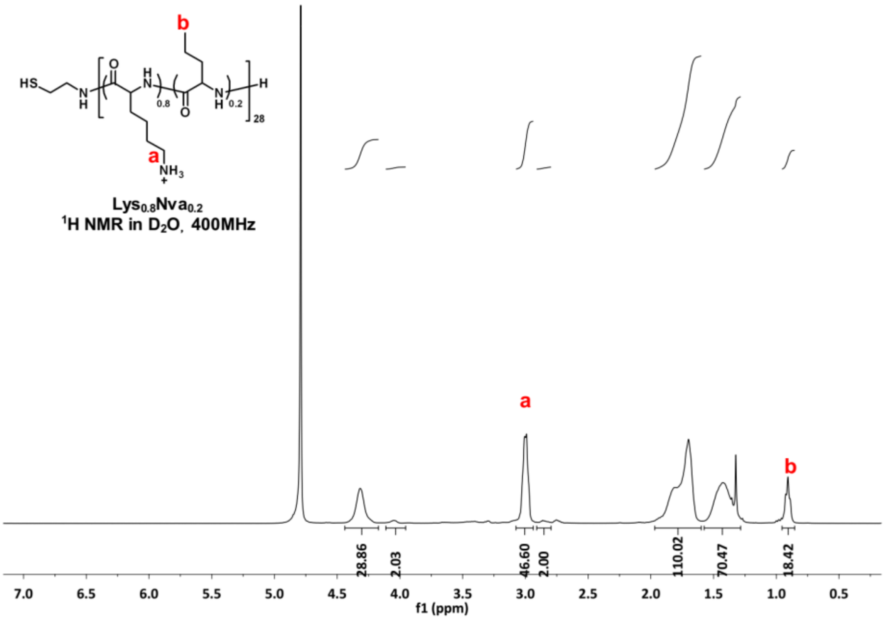

Figure S14. ^{1}H NMR spectrum of $Lys_{0.8}Nva_{0.2}$ in D_2O, 400 MHz.

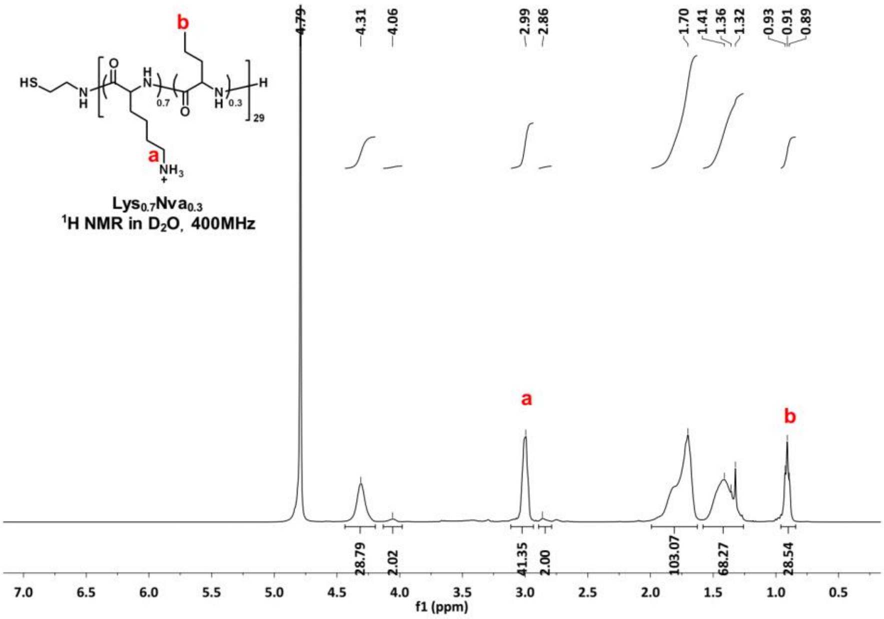

Figure S15. ^{1}H NMR spectrum of $Lys_{0.7}Nva_{0.3}$ in D_2O, 400 MHz.

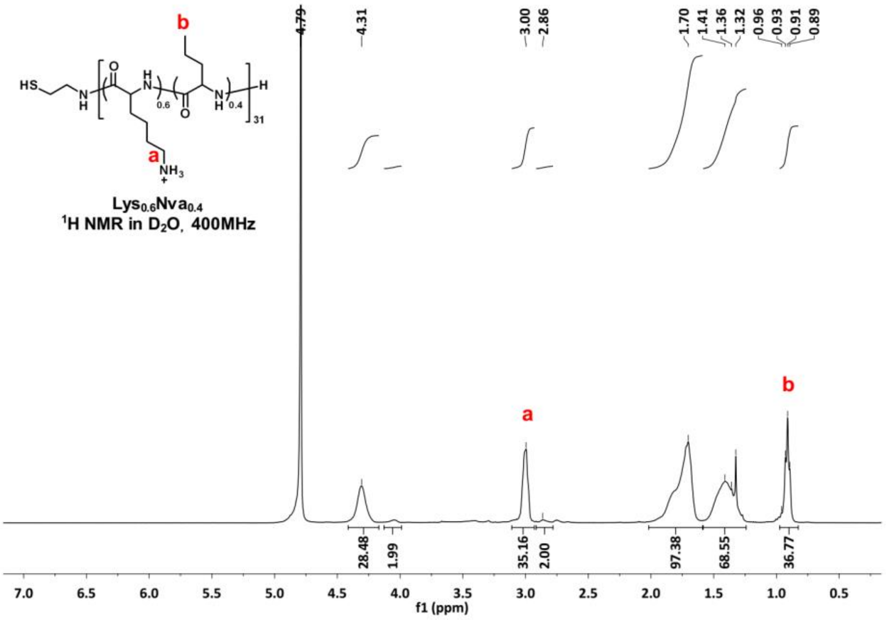

Figure S16. ^{1}H NMR spectrum of $Lys_{0.6}Nva_{0.4}$ in D_2O, 400 MHz.

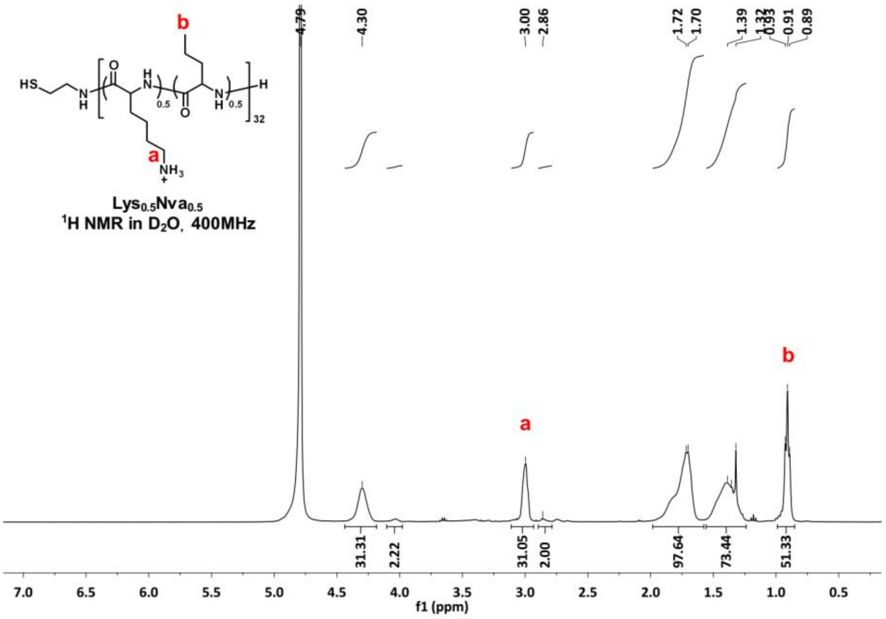

Figure S17. ^{1}H NMR spectrum of $Lys_{0.5}Nva_{0.5}$ in D_2O, 400 MHz.

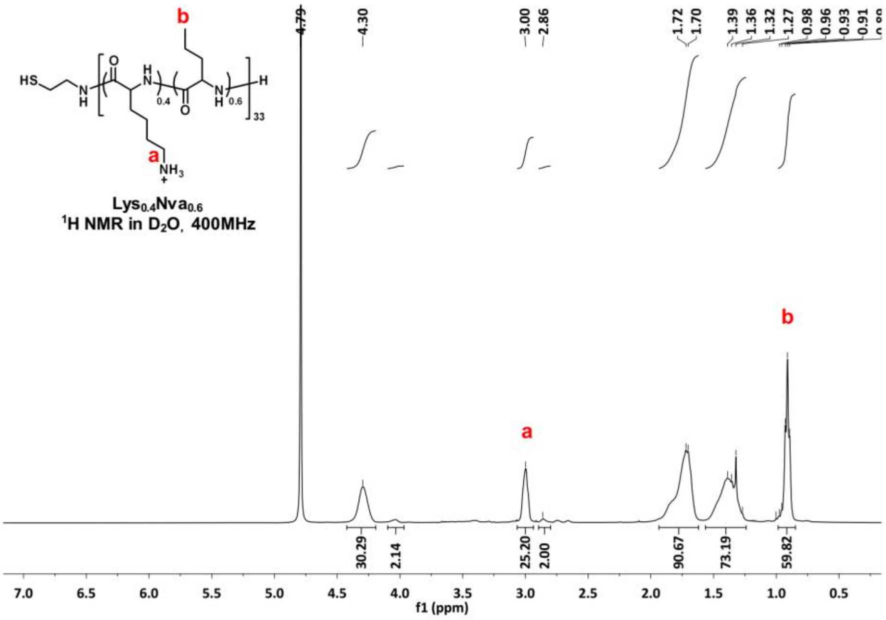

Figure S18. ^{1}H NMR spectrum of $Lys_{0.4}Nva_{0.6}$ in D_2O, 400 MHz.

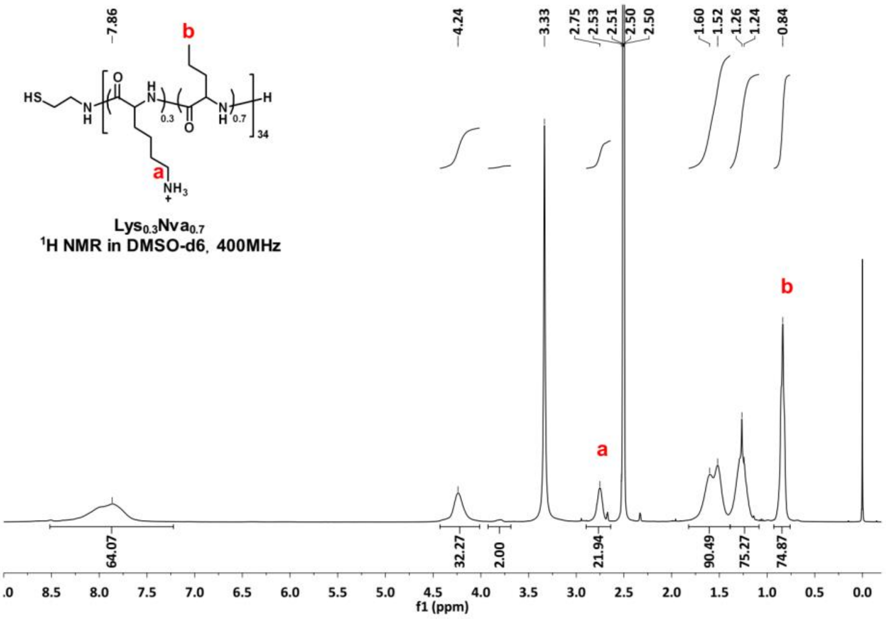

Figure S19. ^{1}H NMR spectrum of $Lys_{0.3}Nva_{0.7}$ in DMSO-*d*6, 400 MHz.

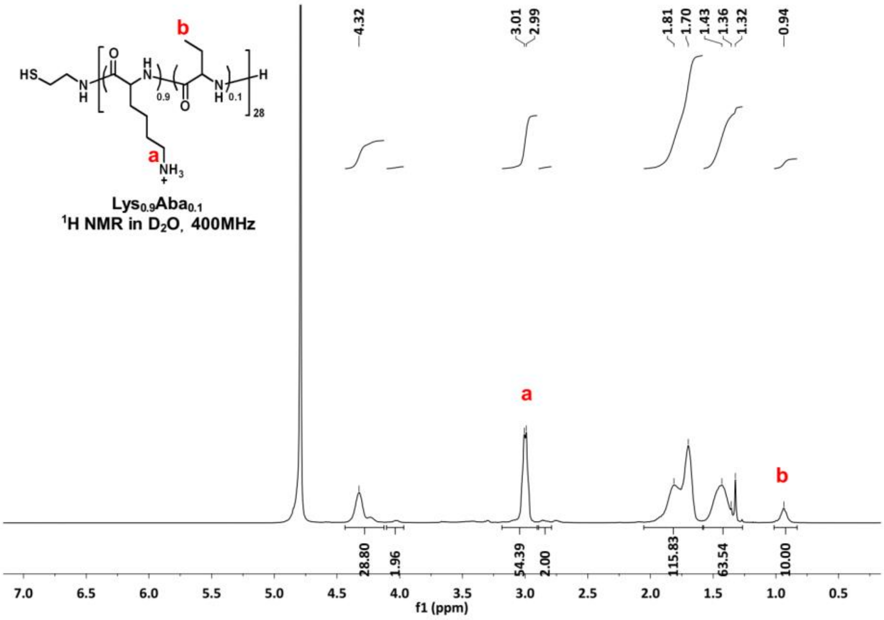

Figure S20. ^{1}H NMR spectrum of $Lys_{0.9}Aba_{0.1}$ in D_2O, 400 MHz.

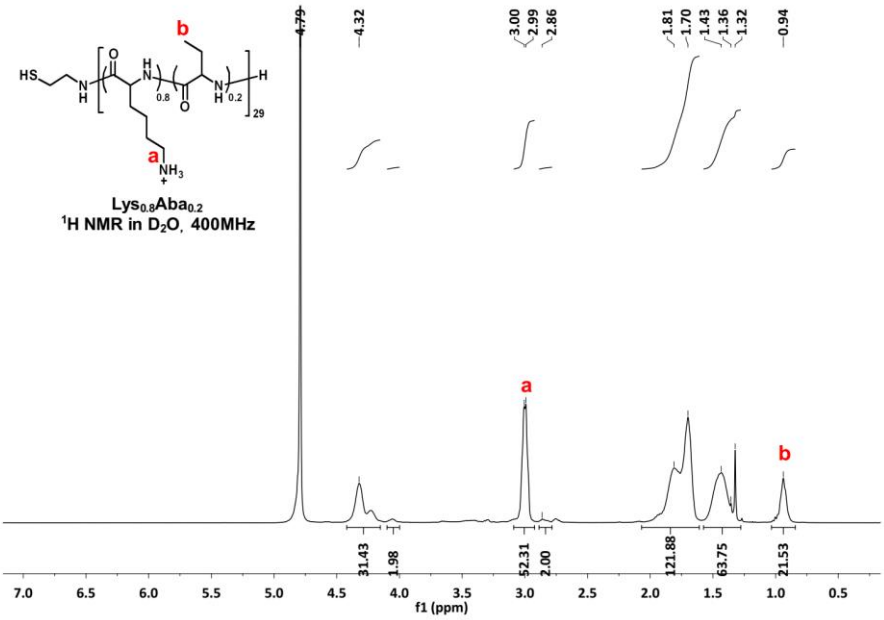

Figure S21. ^{1}H NMR spectrum of $Lys_{0.8}Aba_{0.2}$ in D_2O, 400 MHz.

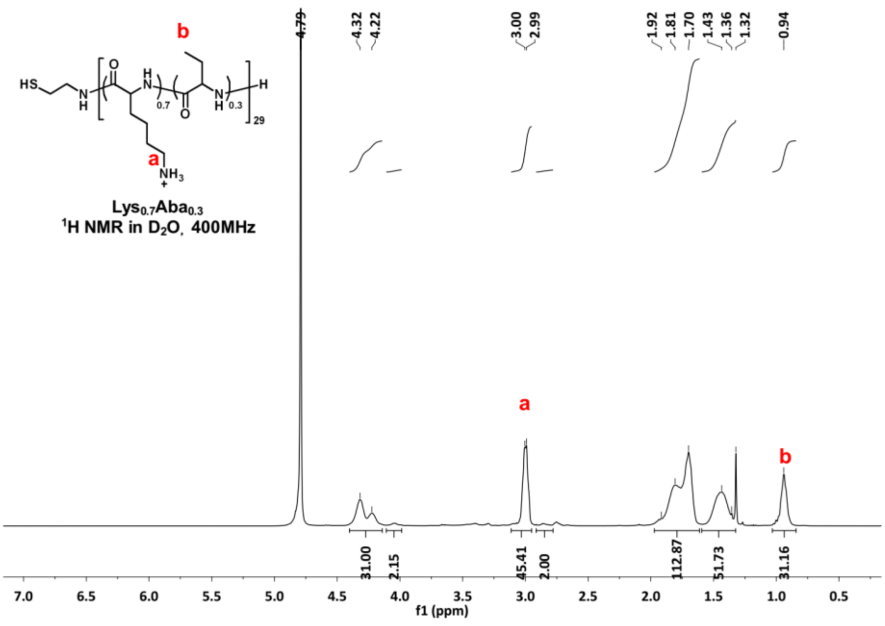

Figure S22. ^{1}H NMR spectrum of $Lys_{0.7}$ $Aba_{0.3}$ in D_2O, 400 MHz.

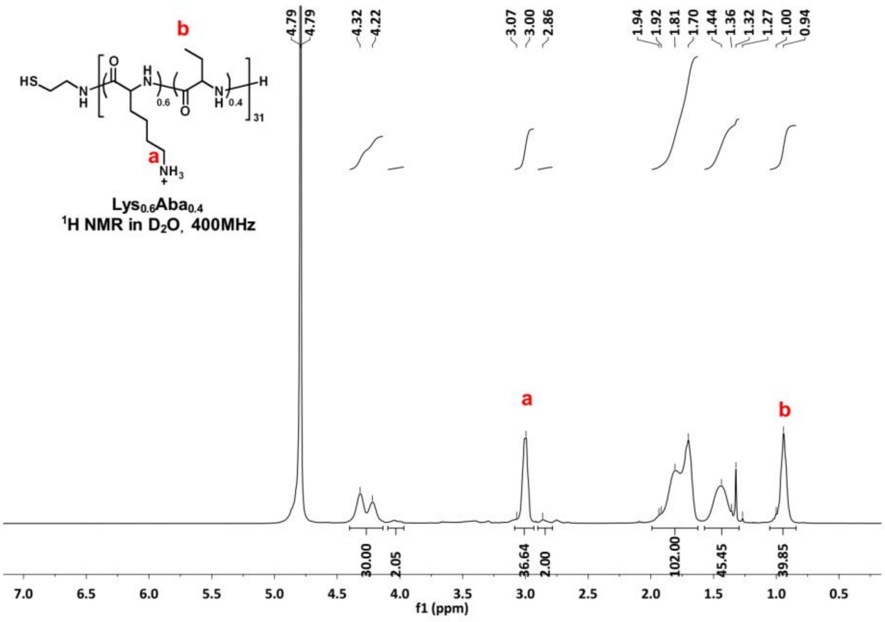

Figure S23. ^{1}H NMR spectrum of $Lys_{0.6}Aba_{0.4}$ in D_2O, 400 MHz.

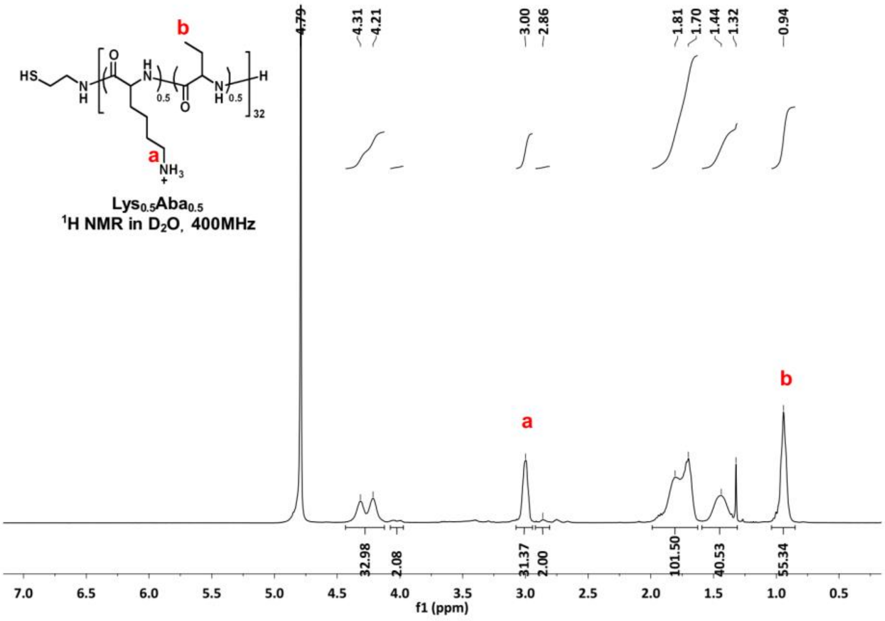

Figure S24. ^{1}H NMR spectrum of $Lys_{0.5}Aba_{0.5}$ in D_2O, 400 MHz.

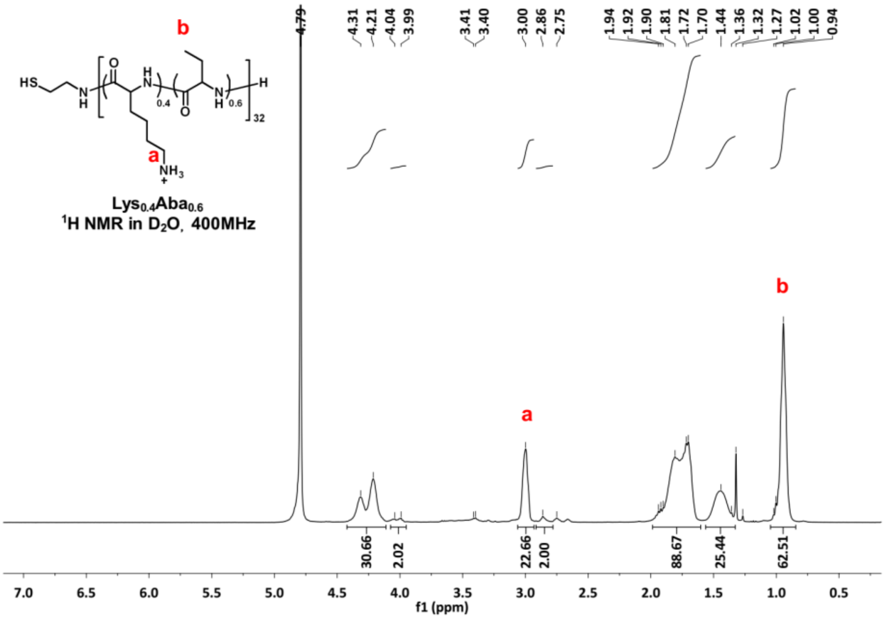

Figure S25. ^{1}H NMR spectrum of $Lys_{0.4}Aba_{0.6}$ in D_2O, 400 MHz.

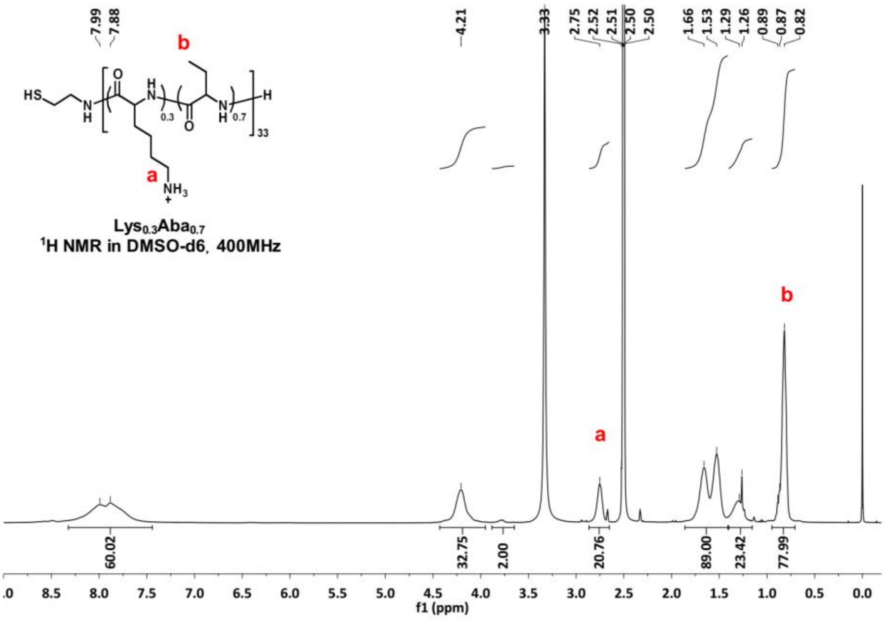

Figure S26. ^{1}H NMR spectrum of $Lys_{0.3}Aba_{0.7}$ in DMSO-*d*6, 400 MHz.

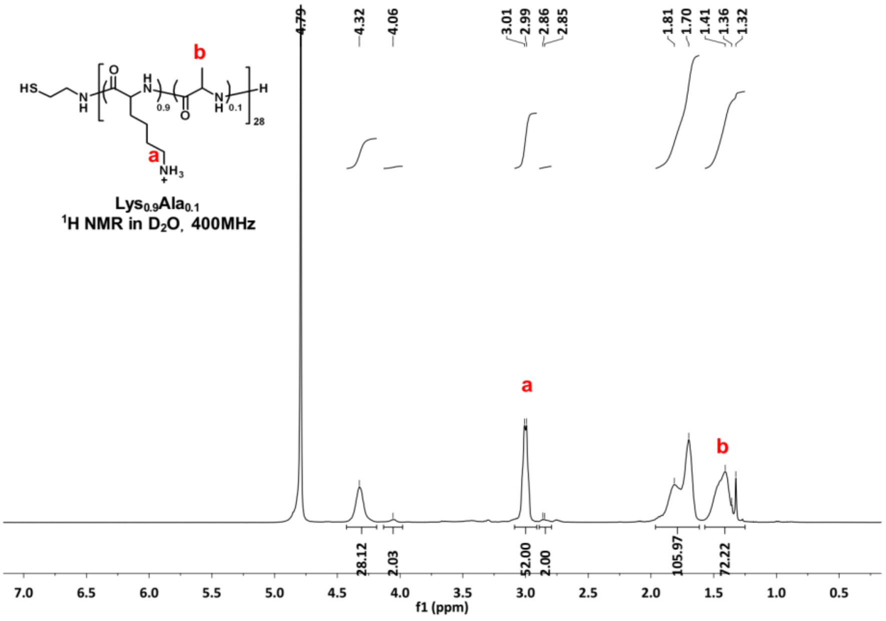

Figure S27. ^{1}H NMR spectrum of $Lys_{0.9}Ala_{0.1}$ in D_2O, 400 MHz.

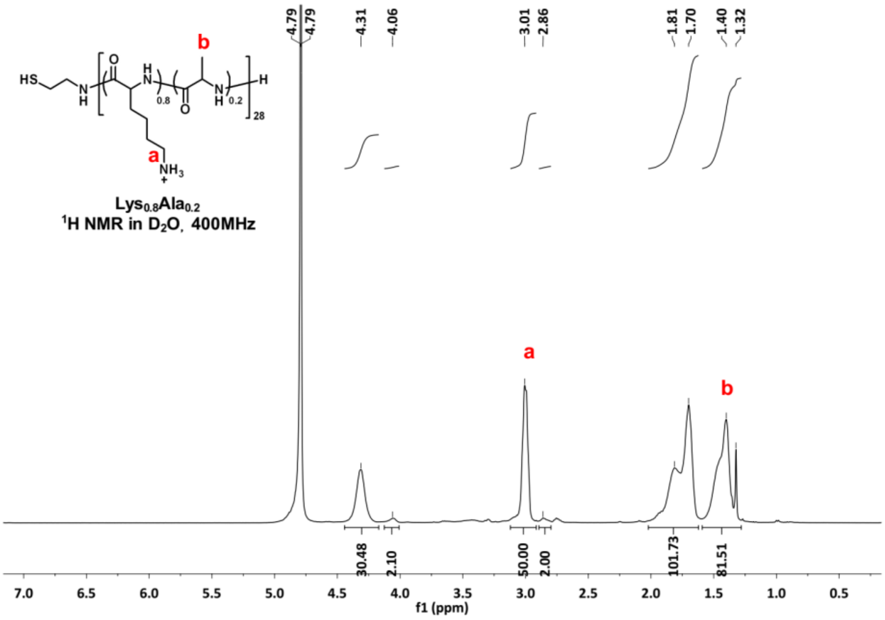

Figure S28. ^{1}H NMR spectrum of $Lys_{0.8}Ala_{0.2}$ in D_2O, 400 MHz.

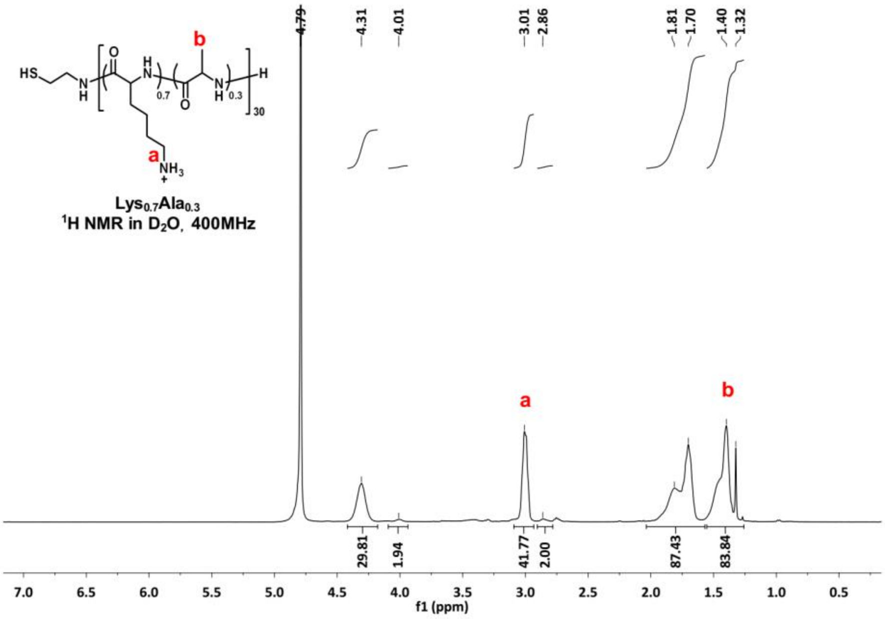

Figure S29. ^{1}H NMR spectrum of $Lys_{0.7}Ala_{0.3}$ in D_2O, 400 MHz.

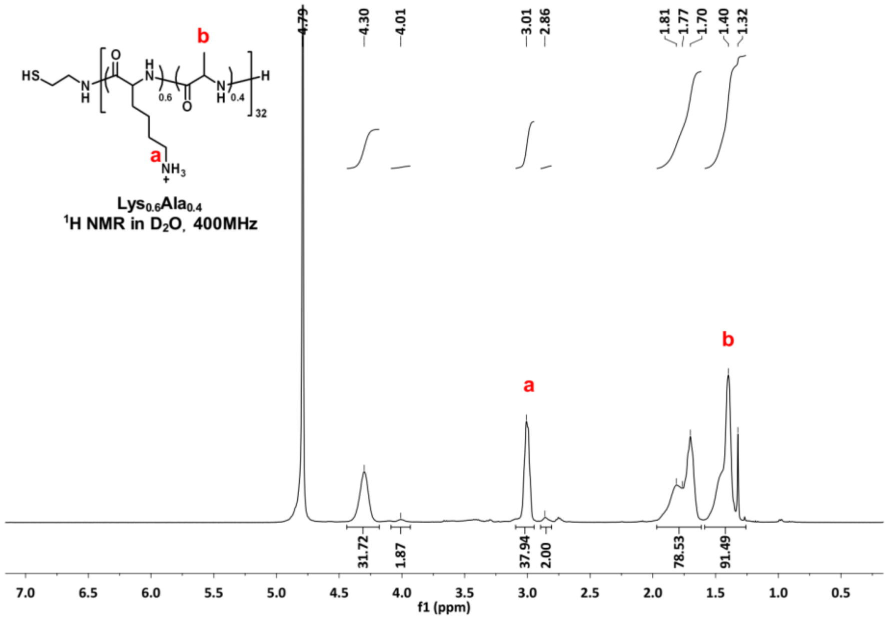

Figure S30. ^{1}H NMR spectrum of $Lys_{0.6}Ala_{0.4}$ in D_2O, 400 MHz.

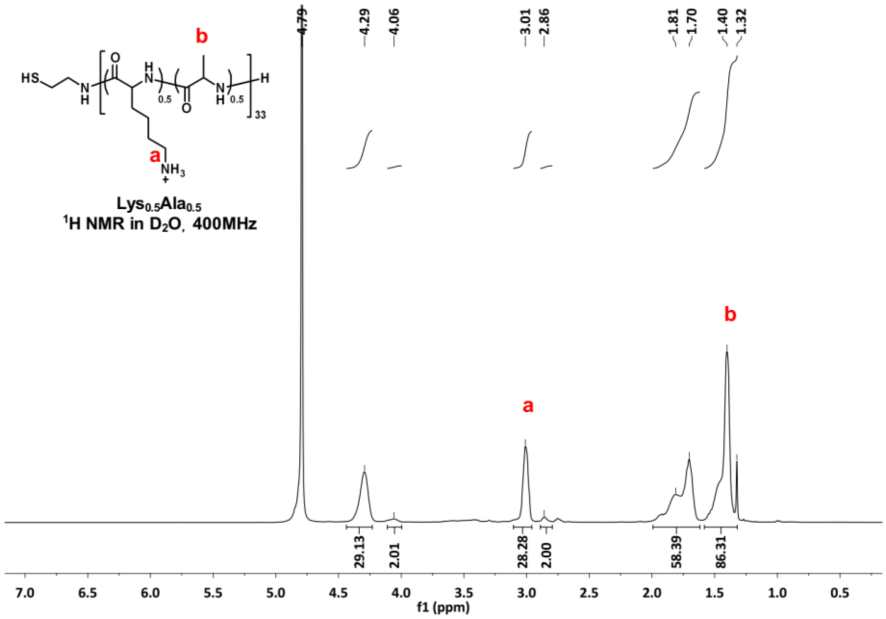

Figure S31. ^{1}H NMR spectrum of $Lys_{0.5}Ala_{0.5}$ in D_2O, 400 MHz.

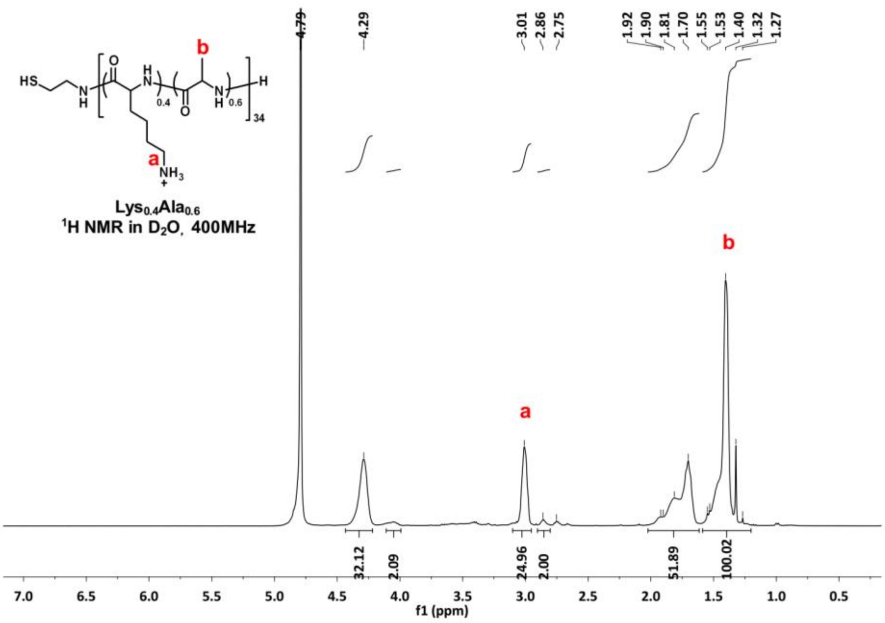

Figure S32. ^{1}H NMR spectrum of $Lys_{0.4}Ala_{0.6}$ in D_2O, 400 MHz.

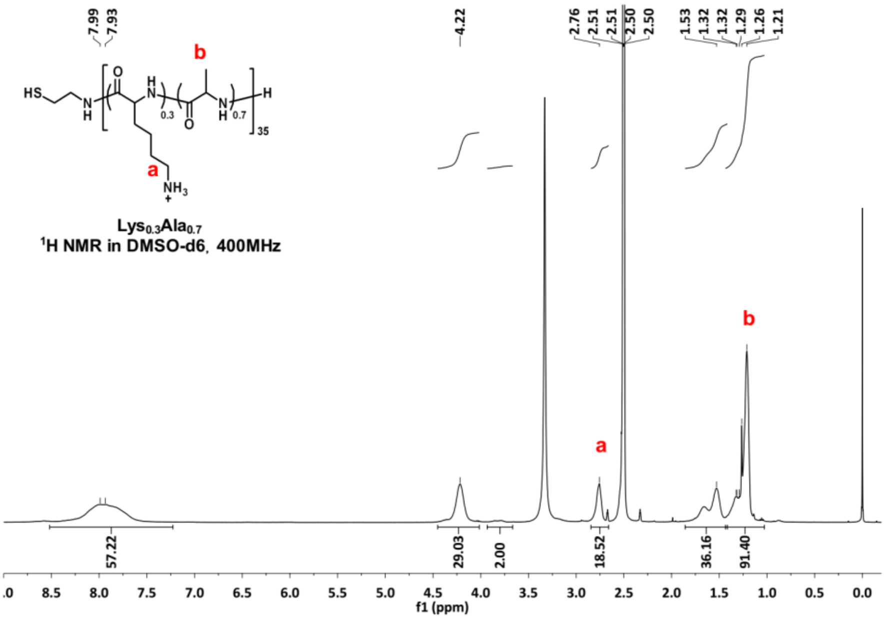

Figure S33. ^{1}H NMR spectrum of $Lys_{0.3}Ala_{0.7}$ in DMSO-*d*6, 400 MHz.

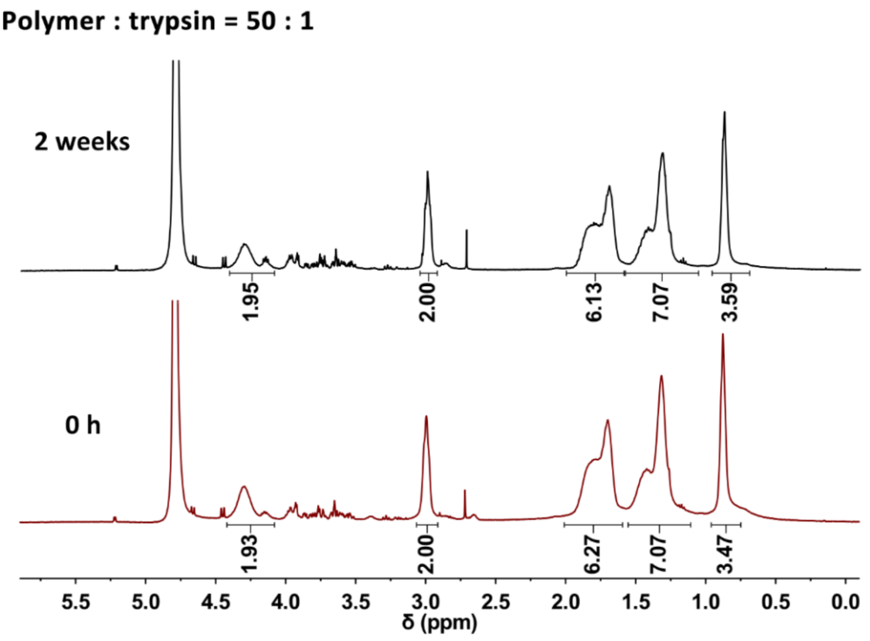

Figure S34. ^{1}H NMR spectra of $Lys_{0.4}Nle_{0.6}$ before and after 2 weeks incubation with trypsin (polymer : trypsin = 50 : 1, w/w) in D_2O, 400 MHz.

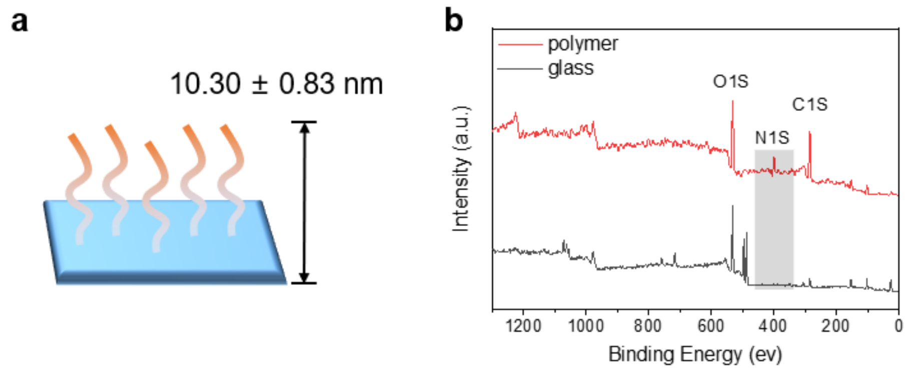

Figure S35. Characterization of polymer modification onto the glass surface using ellipsometry (a) and XPS (b).

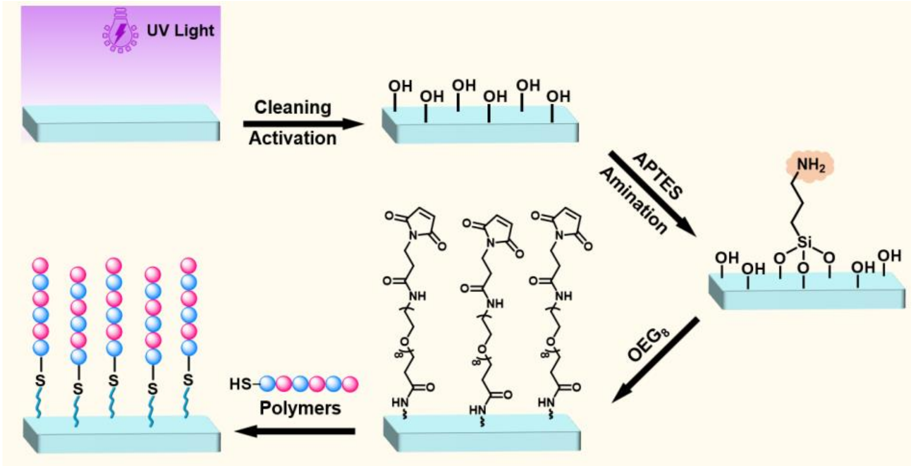

Scheme S5. Schematic illustration of thiol-terminated polymers-modified glass surface.

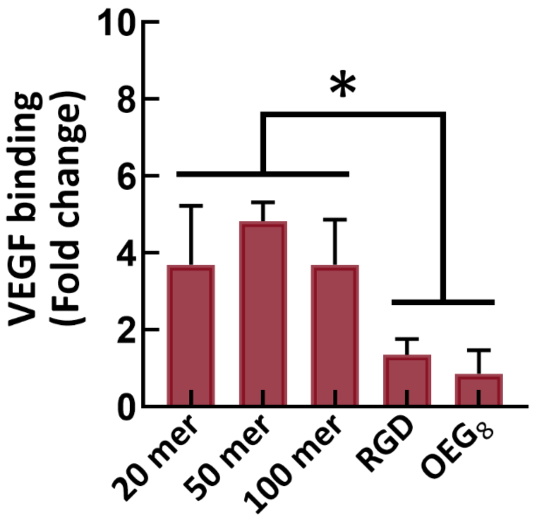

Figure S36. GF binding ability to $Lys_{0.4}Nle_{0.6}$ copolymer with different chain length as evaluated from GF adsorption study (*$p < 0.05$).

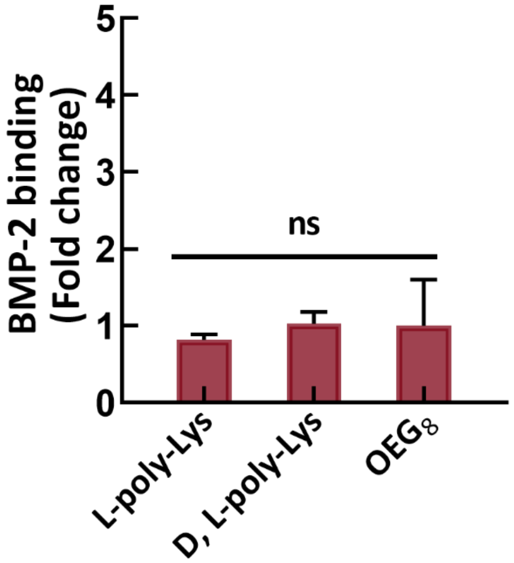

Figure S37. GF binding ability to poly-Lys with different chirality as evaluated from GF adsorption study. No significant difference was observed.

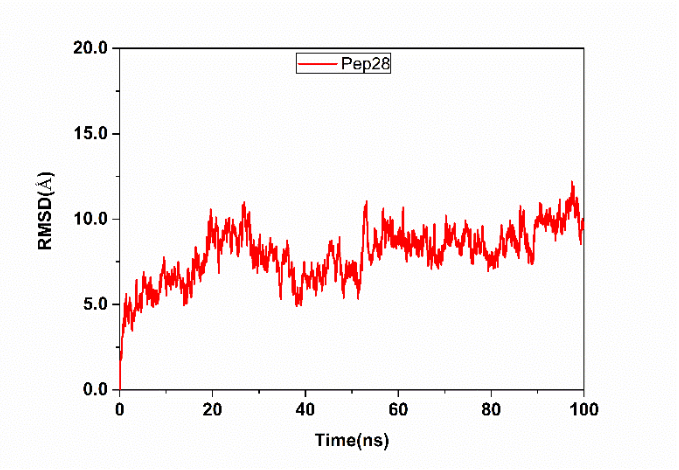

Figure S38. System flexibility analysis of Lys-Nle peptide.

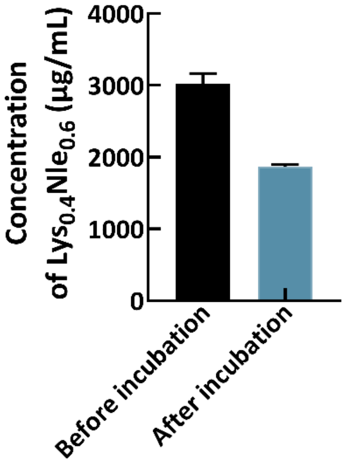

Figure S39. Concentration of $Lys_{0.4}Nle_{0.6}$ in the solution before and after incubation with the Gel scaffold. The reduced concentration of amino acid copolymer solution after incubation with the Gel scaffold indicated the success of copolymer immobilization onto the Gel scaffold.

Table S1. GPC analysis of AA copolymers at the NHBoc protected stage on the relative number average molecular weight (*Mn*), dispersity index (*Đ*), and degree of polymerization (DP).

	GPC characterization											
	M_n	*Đ*	DP	M_n	*Đ*	DP	M_n	*Đ*	DP	M_n	*Đ*	DP
**x:y**	Boc-DL-Lys:DL-Nle			Boc-DL-Lys:DL-Nva			Boc-DL-Lys:DL-Aba			Boc-DL-Lys:DL-Ala		
**9:1**	5950	1.20	28	5930	1.20	28	5920	1.20	28	5890	1.19	28
**8:2**	5750	1.20	28	5650	1.20	28	5730	1.20	29	5570	1.18	28
**7:3**	5820	1.20	30	5520	1.19	29	5330	1.18	29	5390	1.17	30
**6:4**	5740	1.19	31	5440	1.19	31	5240	1.18	31	5380	1.18	32
**5:5**	5250	1.18	30	5250	1.18	32	4960	1.17	32	5010	1.15	33
**4:6**	5170	1.18	32	4980	1.18	33	4560	1.12	32	4590	1.15	34
**3:7**	4740	1.16	32	4620	1.16	34	4200	1.10	33	4130	1.13	35

Table S2. Price comparison between the amino acid polymer and common peptides.

	**Manufacturer**	**The number of amino acid residues**	**Price**
**Amino acid copolymer**	Our lab	28	$3.3/g[a]
**Peptide**	Synpeptide Biotech	28	$1578.5/g
**Peptide**	GenScript Biotech	28	$1400/g
**Magainin II** (a natural antimicrobial peptide)	Sigma-aldrich	25	$1290/mg

[a] The price of our copolymers is calculated based on chemical and reagent costs, personnel cost and synthesis yield. In the case of Lys_xNle_y, the cost of D,L-Lys NCA and D,L-Nle NCA is $0.5/g and $0.38/g, respectively. The polymerization in one batch can provide over 50g of final polymer (75% yield) in 4 days. The total cost is about $3.3/g including $2.5/g of personnel costs and $0.8/g of chemical and reagent costs.

Table S3. Parameters of primers utilized for detecting gene expression

Gene	Direction	Sequence(5’-3’)
**Col I**	Forward	GGT ATG CTT GAT CTG TAT CTG C
	Reverse	AGT CCA GTT CTT CAT TGC ATT
**OCN**	Forward	CTG ACA AAG CCT TCA TGT CCA A
	Reverse	GCG GGC GAG TCT GTT CAC TA
**Runx2**	Forward	CGG CCC TCC CTG AAC TCT
	Reverse	TGC CTG CCT GGG ATC TGT
**GAPDH**	Forward	GTC GTG GAG TCT ACT GGT GTC
	Reverse	GAG CCC TTC CAC AAT GCC AAA
